# Kisameet Glacial Clay: an Unexpected Source of Bacterial Diversity

**DOI:** 10.1128/mBio.00590-17

**Published:** 2017-05-23

**Authors:** Sarah L. Svensson, Shekooh Behroozian, Wanjing Xu, Michael G. Surette, Loretta Li, Julian Davies

**Affiliations:** a Department of Microbiology and Immunology, University of British Columbia, Vancouver, British Columbia, Canada; b Department of Civil Engineering, University of British Columbia, Vancouver, British Columbia, Canada; c Department of Medicine, McMaster University, Hamilton, Ontario, Canada; Emory University

**Keywords:** *Actinobacteria*, clay mineral, geochemical characteristics, Kisameet, microbiome, antimicrobial activity, microbial communities

## Abstract

Widespread antibiotic resistance among bacterial pathogens is providing the impetus to explore novel sources of antimicrobial agents. Recently, the potent antibacterial activity of certain clay minerals has stimulated scientific interest in these materials. One such example is Kisameet glacial clay (KC), an antibacterial clay from a deposit on the central coast of British Columbia, Canada. However, our understanding of the active principles of these complex natural substances is incomplete. Like soils, clays may possess complex mixtures of bacterial taxa, including the *Actinobacteria*, a clade known to be rich in antibiotic-producing organisms. Here, we present the first characterization of both the microbial and geochemical characteristics of a glacial clay deposit. KC harbors surprising bacterial species richness, with at least three distinct community types. We show that the deposit has clines of inorganic elements that can be leached by pH, which may be drivers of community structure. We also note the prevalence of *Gallionellaceae* in samples recovered near the surface, as well as taxa that include medically or economically important bacteria such as *Actinomycetes* and *Paenibacillus*. These results provide insight into the microbial taxa that may be the source of KC antibacterial activity and suggest that natural clays may be rich sources of microbial and molecular diversity.

## IMPORTANCE

Identifying and characterizing the resident microbial populations (bacteria, viruses, protozoa, and fungi) is key to understanding the ecology, chemistry, and homeostasis of virtually all sites on Earth. The Kisameet Bay deposit in British Columbia, Canada, holds a novel glacial clay with a history of medicinal use by local indigenous people. We previously showed that it has potent activity against a variety of antibiotic-resistant bacteria, suggesting it could complement our dwindling arsenal of antibiotics. Here, we have characterized the microbiome of this deposit to gain insight into what might make the clay antibacterial. Our analyses suggest that the deposit contains a surprising diversity of bacteria, which live in at least three distinct environments. In addition, the clay harbors bacteria that may have interesting potential as biocontrol/bioremediation agents or producers of novel bioactive compounds.

## INTRODUCTION

Microbes are essential components of most of the environments on Earth. They survive, and even thrive, in a variety of niches and factor centrally in metabolic pathways that shape geologic, ecological, and human health. A holistic understanding of how microbes cycle different elements, such as carbon in the soil and oceans, is essential to understanding global homeostatic processes (1), and the microbiome is likewise being accepted as an essential component of the human system. An understanding of microbial diversity is therefore central to environmental, evolutionary, and biomedical sciences. Cultivation-independent molecular surveys have revealed the extent of this diversity and have identified some factors that may influence microbial community structures. This knowledge is revolutionizing our understanding of the contribution of the microbiome to human health and disease (2, 3). However, these studies are only now beginning to reveal the phylogeny, gene content, and phenotypic potential of the so-called microbial dark matter, and comprehensive surveys of microbes in novel environments are necessary (1, 4).

There is also a pressing, if not desperate, need for new strategies to replace or complement existing antimicrobial agents in the face of ever-increasing (multi)antibiotic resistance (5). Much research is focused on identifying “wild” microbes that might produce novel antimicrobials (6–8). However, processing new candidates through the research and regulatory pipelines is slow. As such, “historical” agents are increasingly being investigated in modern empirical studies for treatment of infectious disease. The medicinal applications of natural substances such as clays, muds, and other natural terrestrial products have been known for thousands of years. More recently, the use of poultices of French green clay for treatment of *Mycobacterium ulcerans* skin infections in Africa by the humanitarian Line Brunet de Courssou has renewed interest in the antibacterial potential of clays (9, 10).

Clays are a diverse group of economically important natural materials, made up of clay minerals—silicates with a repeating layer structure and a small particle size (<2 μm) (11). Their small particle size and large surface area provide novel adsorptive and ion-exchange characteristics, which may underlie their therapeutic and cosmetic properties (12). Pioneering work has demonstrated the broad spectrum of activity of several natural mineral clays *in vitro* (13–16; reviewed in reference 17). The mechanism(s) of action of different clays appears diverse and/or multifactorial and may include pH and redox buffering of metal ion toxicity (Fe^2+^ and Al^3+^), adsorption and release of compounds in clay interlayers, absorption of micronutrients required for bacterial growth, or proliferation of bacterial species that produce antibacterial compounds (14, 18, 19).

Kisameet glacial clay (KC), located in a deposit on the northwestern coast of British Columbia, Canada, has been used historically by the local Heiltsuk First Nation for therapeutic purposes. KC has potent antibacterial activity against multidrug-resistant ESKAPE (*Enterococcus faecium*, *Staphylococcus aureus*, *Klebsiella pneumoniae*, *Acinetobacter baumannii*, *Pseudomonas aeruginosa* and *Enterobacter* species) pathogens (20), which are common nosocomial species that frequently escape current antibiotic treatments (21). KC is also strongly active against extensively resistant and multidrug-resistant opportunistic pathogens, such as members of the *Burkholderia cepacia* complex, isolated from cystic fibrosis patients (S. Behroozian, J. E. A. Zlosnik, and J. Davies, submitted for publication). The clay is also active against fungal pathogens such as *Candida albicans* and *Cryptococcus neoformans* (S. Behroozian, S. L. Svensson, W. Xu, L. Li, and J. Davies, unpublished data). KC is relatively rich in iron (22), and therefore, pH-dependent metal ion toxicity, as described for other clays (13, 14), may underlie some of its activity (Behroozian et al., unpublished). Active fractions can also be extracted with organic solvents (J. Tan and J. Davies, unpublished data), suggesting that the mechanism of action might be multifactorial. Much remains to be identified about the mechanism of antimicrobial activity of KC to facilitate the production of consistent, safe, and potent preparations for future medical applications.

The overall expressed properties of many natural products may be the result of biochemical interactions with their resident microbiota. A comprehensive microbiome study of a clay deposit *in situ* has not yet, to our knowledge, been reported. Previous microbiological studies of clays have been limited to small sample sizes of processed samples or clay-rich soils. Here, we describe investigations of the bacterial communities of the KC deposit by 16S rRNA metagenome characterization from five vertical cores. The deposit has three general types of bacterial community, which correlate with depth and pH. We found surprisingly high bacterial species diversity, with a dominance of *Betaproteobacteria*. Identification of bacterial taxa that make up the dominant core of each community revealed differences in the distribution of putative iron-oxidizing *Gallionella* spp. Bacterial community structure did not correlate with antibacterial activity. However, we found evidence that the KC microbiome includes potentially valuable species, such as *Actinobacteria*. This initial microbiological survey of a novel environment provides a framework for understanding the basis for the unique properties of KC.

## RESULTS

### Description of the Kisameet Bay deposit and experimental design.

**FIG 1:**
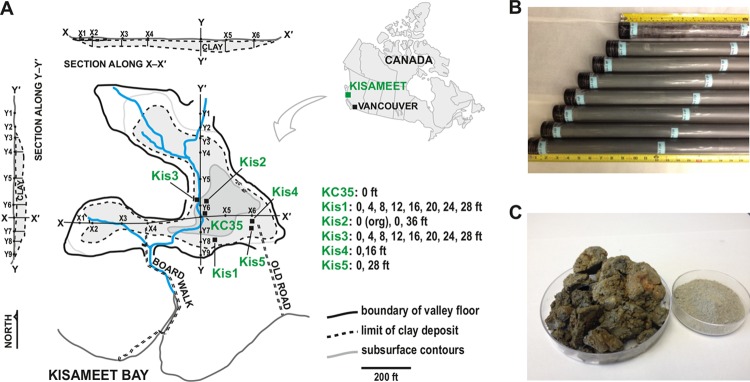
The Kisameet Bay glacial clay deposit, spatial description of core sampling, and physical appearance of KC. (A) The approximate location of Kisameet Bay on King Island on the central coast of British Columbia, ∼450 km northwest of Vancouver, Canada, is shown in the top right inset. The topographical layout of the deposit showing the approximate locations of the five vertical core samples (Kis1 to Kis5) and KC35 makes up the main part of panel A. The KC deposit map is simplified/modified from reference 23. (B) Examples of KC vertical core samples (Kis5), showing the green-gray color of the clay *in situ*, as well as organic overburden present in some shallow (i.e., 0 to 4 ft) samples from the top of the deposit. (C) KC following exposure to the air, showing reddish-brown areas, as well as a dried and powdered sample.

Kisameet glacial clay is found in a dry-land (i.e., nonmarine) deposit on the Central Coast of British Columbia in Canada, 450 km northwest of Vancouver (51°58′20″ N/127°52′50″ W) (23, 24) (Fig. 1A, map in the top right corner). The deposit covers ~2 ha and is estimated to contain 181,000 tons of clay in a depression sitting on sand, gravel, and bedrock to a maximum depth of 40 ft (Fig. 1A). The clay itself is Quaternary in age, resulting from glacial expansion and retreat during past ice ages but sits upon gray and black schist and gneiss of the Jurassic to Tertiary Coast Plutonic Complex (23). A layer of organic overburden of up to 2 meters thick covers the deposit, and the clay itself is dark greenish-gray when moist and light gray when dry (Fig. 1B). Areas of reddish-brown material can also be present, especially upon exposure to air (Fig. 1C). The KC deposit remains mostly untouched, but it was drilled in 1946, and samples were submitted to the British Columbia Mines Branch. About 25 tons of clay were sold as a natural pharmaceutical and cosmetic. Initial mineralogical studies indicated that KC is an iron-rich (4.5% by weight) bentonite clay, which is fine-grained (54.6% with a grain diameter of 1.7 to 3 μm and 30.7% with a diameter of <1.7 μm) (22, 23).

Five vertical cores were extracted from the deposit at five locations (Kis1 to Kis5 [Fig. 1A]). Because the depth of the deposit is variable, Kis1 extended from 0 ft to ~32 ft, for example, whereas the bottom of the Kis4 core was only ~20 ft below the surface. Each core was then divided into 4-ft-long sections and labeled according to the depth at the top of the section. For example, the Kis3 core was separated into samples Kis3-0 (0 ft ≤ depth < 4 ft), Kis3-4 (4 ft ≤ depth < 8 ft), Kis3-8 (8 ft ≤ depth < 12 ft), and so on, where the first number indicates the core and the second number indicates the depth. Twenty-three of these sections were selected for physicochemical and microbiome characterization (listed in Table S1 in the supplemental material). Two of the samples, Kis3-0 Org and Kis5-0, consisted primarily of organic material from overburden (Kis3-0 was separated into “organic” and “clay” fractions). We also analyzed KC35, a sample from the surface that has been previously shown to have strong antimicrobial activity (20).

10.1128/mBio.00590-17.6 TABLE S1  Library and read statistics for next-generation sequencing. Download TABLE S1, DOCX file, 0.03 MB. Copyright © 2017 Svensson et al. 2017 Svensson et al. This content is distributed under the terms of the Creative Commons Attribution 4.0 International license.

### Physicochemical characteristics of the deposit.

We first performed a general characterization of the physicochemical properties of KC. Mineralogical analysis of selected core samples showed that KC contains phyllosilicates such as biotite (~3 to 15%) and relatively small amounts of clay minerals such as illite (~5%) and clinochlore (~5 to 13%) (Table S2). We did not observe any major differences between the selected core samples and KC35. Likewise, analysis of acid-digested elements in KC core samples and elemental analysis of bulk clay did not reveal major variations (Table S3) but confirmed previous observations of significant amounts of Fe (~10^4^ mg/kg). Because speciation could presumably affect the antibacterial activity of toxic metals, including Fe, we also determined the redox potential of each sample (Table S3). The measured potential of the core samples varied relatively widely from +19 mV to a maximum of +373 mV, with the redox of KC35 being +427 mV.

10.1128/mBio.00590-17.7 TABLE S2  Mineralogical composition of selected KC core samples. Download TABLE S2, DOCX file, 0.03 MB. Copyright © 2017 Svensson et al. 2017 Svensson et al. This content is distributed under the terms of the Creative Commons Attribution 4.0 International license.

10.1128/mBio.00590-17.8 TABLE S3  Acid-digested elemental composition of KC core sample bulk clay. Download TABLE S3, DOCX file, 0.04 MB. Copyright © 2017 Svensson et al. 2017 Svensson et al. This content is distributed under the terms of the Creative Commons Attribution 4.0 International license.

**FIG 2:**
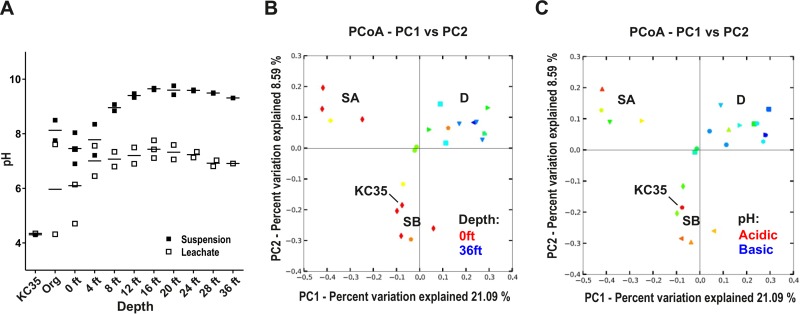
KC communities cluster into three types (SA, SB, and D) according to depth and pH. (A) The pH of KC samples varies with depth. The pH of either fresh aqueous suspensions or prepared aqueous leachates of each sample was measured. The measurement for each individual sample, as well as the mean, is plotted. (B and C) Principal-coordinate analysis of core sample communities. Communities for each core sample were clustered using principal-coordinate analysis (PCoA) of unweighted UniFrac distances obtained from 16S rRNA amplicon sequencing. In panel B, symbols representing each KC sample are colored according to a gradient representing depth. In panel C, symbols are colored according to a gradient representing their aqueous leachate pH (acidic [pH ~4] to basic [pH ~9]). PC1, principal component 1.

In contrast, comparison of the pH of core sample suspensions revealed more marked differences. While previous studies suggested that KC is mildly alkaline (pH ~8) (15), the low pH of KC35 we measured previously (20) suggests that some samples could be markedly more acidic. Measurement of different preparations of KC core samples (fresh aqueous suspensions, aqueous leachates, or suspensions of clay that had been stored exposed to air for approximately 1 month) suggested that the clay pH can vary depending on (i) depth of the sample in the deposit and (ii) exposure to air. First, the pH of fresh KC suspensions revealed a strong trend with depth (Fig. 2A and Table S4), with the pH of clay from greater depths significantly higher than for samples from depths of 0 to 8 ft (pH 9.5 versus pH 8.0; *P* < 0.0001). Also, while the pH of prepared aqueous leachates showed the same positive correlation between increasing pH and depth, we also noted that in general, aqueous leachates showed a lower pH than their suspension counterparts (Fig. 2A). This may reflect a lowering of pH during the 24 h of incubation under aeration during leachate preparation. In support of this, remeasurement of suspension pHs a few months after core cutting resulted in markedly higher levels of acidity. In addition, the pH of KC35, which was harvested at least 2 years before the core samples and stored at 4°C, had an extremely low suspension pH of 4.3. Together, this suggests that the pH of KC can vary markedly depending on depth-related factors, as well as exposure to air.

10.1128/mBio.00590-17.9 TABLE S4  Physicochemical characteristics of KC core samples. Download TABLE S4, DOCX file, 0.03 MB. Copyright © 2017 Svensson et al. 2017 Svensson et al. This content is distributed under the terms of the Creative Commons Attribution 4.0 International license.

### KC microbiomes cluster into three distinct community types that correlate with depth and pH.

DNA was extracted from each core sample, and community profiling was performed by sequencing 16S rRNA amplicons. We identified 5,032 unique operational taxonomic units (OTUs) (3% genetic distance definition), with a range of 281 (Kis1-28) to 2,410 (Kis3-0 Org). Principal-coordinate analysis (PCoA) of unweighted UniFrac distances (25) was performed to compare beta diversity of KC communities. The first two components accounted for 21.09% and 8.59% of the difference between the communities (Fig. 2B). The KC communities grouped into three clusters (Table S1). The PCoA plot was first overlaid with depth information (i.e., 0 ft, 4 ft, and so on). This suggested that two of the clusters included most of the shallower samples (0 to 8 ft), as well as KC35 (also from the surface) (Fig. 2B). We therefore refer to these “shallow” clusters as SA and SB. In contrast, the third cluster, termed D (“deep/interior”), included mostly samples from 12 ft and below, although some surface samples, such as Kis1-0 and Kis3-4, were in this cluster. For surface (0 to 8 ft) samples in clusters SA and SB, there may also be an effect of the horizontal location of the sample. For example, cluster SA samples were all from Kis1, Kis3, and Kis5 (edge of the deposit [Fig. 1A]), whereas cluster SB samples were from Kis2 and Kis4 (closer to the interior). When the leachate pH was overlaid onto PCoA analyses, we saw a trend reminiscent of depth analysis, where more acidic samples were part of either cluster SA or SB (Fig. 2C). Statistical analyses also suggested that both cluster SA and cluster SB samples had a significantly lower pH than cluster D (*P* = 0.047 and *P* = 0.0073, respectively), while the pH of clusters SA and SB was not significantly different (*P* > 0.5). This suggests that a depth-driven cline in pH affected the microbiome of KC. The KC deposit has a significant organic overburden of macroflora (Fig. 1B), which might decrease the pH at the surface. The KC deposit therefore harbors several different bacterial communities, likely shaped by environmental parameters that change with depth.

### KC harbors rich bacterial diversity that is dominated by *Betaproteobacteria.*

**FIG 3:**
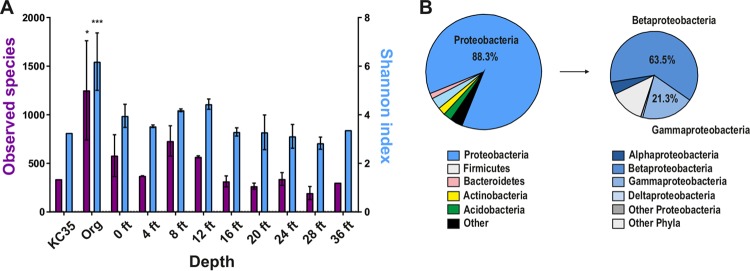
Bacterial diversity of KC core samples. (A) Mean measures of alpha diversity (observed species [OTUs] and Shannon index) for KC35, core samples composed mostly of organic (Org) material (Kis3-0 Org and Kis5-0), and clay samples from different depths as identified by 16S rRNA amplicon sequencing. The mean ± standard error of the mean (SEM) (error bar) for each indicated sample group for OTUs detected at the same sequencing depth (41,294 reads), not including doubleton and singleton OTUs, are plotted. The values for Org samples that were significantly different from the values for all other samples by Student’s *t* test are shown by asterisks: *, *P* ≤ 0.05; ***, *P* ≤ 0.001. (B) Phylum- and class-level analysis of total assigned reads for all KC samples. OTUs with only one or two reads are not included in this analysis.

As noted above, we identified more than 5,000 unique OTUs, with a range of 281 (Kis1-28) to 2,410 (Kis3-0 Org). Alpha rarefaction analysis of the sequence data showed that our survey is likely an underestimation of the diversity of some samples (Fig. S1). Nonetheless, we compared the bacterial diversity of each KC sample (observed species and Shannon index) (26) at the same sequencing depth, not including singleton and doubleton OTUs (Fig. 3A). The KC deposit has a layer of overburden (Fig. 1B), and samples that were composed of almost entirely organic material (Kis3-0 Org and Kis5-0; greater than 90% amorphous by X-ray diffraction [XRD]) had a higher mean observed species (mean 1,251 OTUs versus 431 for clay samples; *P* = 0.0015) and Shannon index (6.2 versus 3.6 for clay samples; *P* = 0.0003) metrics. Among the clay samples, there was also a minor trend toward lower diversity measures at greater depths (Fig. 3A). We next identified the major bacterial phyla in KC samples. Most of the reads (88.3%) were assigned to *Proteobacteria* OTUs. Other significant phyla identified included *Bacteroidetes* (3.2%), *Actinobacteria* (2.4%), *Acidobacteria* (1.8%), and *Firmicutes* (1.3%) (Fig. 3B). Further separation of the *Proteobacteria* into constituent classes revealed that approximately 63.5% of reads for this phylum could be assigned to *Betaproteobacteria*, followed by *Gammaproteobacteria* (21.3%), *Alphaproteobacteria* (4.0%), and *Deltaproteobacteria* (0.4%). These results are consistent with other surveys of bacteria in soil environments, which have shown that most are dominated by *Proteobacteria* (27). *Acidobacteria* and *Actinobacteria* are also common in soils. Thus, KC harbors a surprising number of different bacterial species, with a large contribution from *Proteobacteria*.

10.1128/mBio.00590-17.2 FIG S1  Alpha rarefaction for sequencing data. Download FIG S1, PDF file, 0.1 MB. Copyright © 2017 Svensson et al. 2017 Svensson et al. This content is distributed under the terms of the Creative Commons Attribution 4.0 International license.

### The core bacterial communities of KC.

In general, *Betaproteobacteria* and *Gammaproteobacteria* species dominated the sequences obtained for all KC samples (Fig. 3B). However, three distinct communities may be present in KC (Fig. 2B and C). To identify taxa that might reveal differences between these communities and/or their environments, we defined a core bacterial community (CBC) for each of the three PCoA clusters. We defined CBCs based on common membership (i.e., an OTU was part of the cluster CBC if at least one sequence read was present in all samples of that cluster). CBC OTUs were also limited to those that made up at least 0.1% of the total reads in the cluster being compared. We chose this approach because of limitations in sample harvesting and storage (see Materials and Methods), and thus, we did not take into account the proportion of the community made up by an OTU, as has been done previously (28).

**FIG 4:**
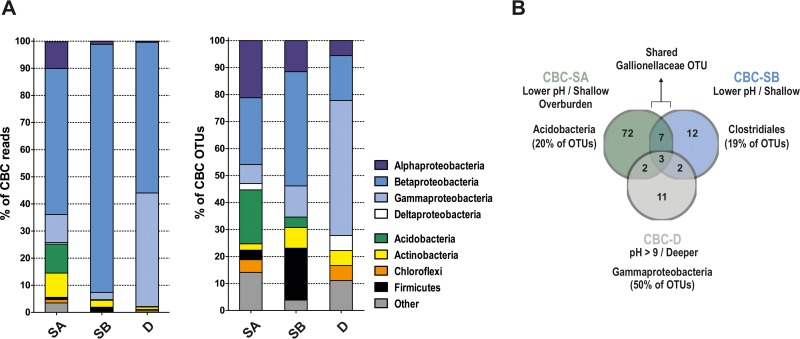
The core bacterial community (CBC) of KC clusters. For each cluster, OTUs were identified. These OTUs had at least one read in each sample designated part of the cluster and that also made up at least 0.1% of all reads for the cluster. (A, left) Distribution of reads among phyla and proteobacterial classes for OTUs determined to be part of the CBC of each cluster (SA, SB, or D). (Right) Distribution of CBC OTUs among different phyla and proteobacterial classes. Complete OTU listings for each CBC are shown in Table S5 in the supplemental material. (B) Shared and distinct OTUs of cluster CBCs. Details of shared OTUs are listed in Table S5.

Under this definition, only a handful of the species we detected (5,032 OTUs) made up the CBC of each KC community (Table S5). For example, the CBC of cluster D, which included 14 of the KC samples, included only 18 OTUs. For comparison between communities, CBC OTUs were separated into major phyla (*Proteobacteria*, *Acidobacteria*, *Actinobacteria*, *Chloroflexi*, or *Firmicutes*). Proteobacterial OTUs were also separated according to class (i.e., *Alpha*-, *Beta-*, *Gammaproteobacteria*, etc.). We then compared the proportion of either (i) reads or (ii) OTUs from each phylum/class between the CBCs (Fig. 4A). *Betaproteobacteria* dominated CBC reads for all three clusters (53.8%, 91.5%, and 55.6% for CBC-SA, CBC-SB, and CBC-D, respectively) (Fig. 4A, left panel). However, while a significant proportion of CBC-SA reads were also assigned to *Acidobacteria* (10.7%), *Gammaproteobacteria* (10.4%), *Alphaproteobacteria* (9.8%), and *Actinobacteria* (9%), CBC-SB was dominated by *Betaproteobacteria*. Finally, reads for CBC-D showed a more even distribution between *Betaproteobacteria* and *Gammaproteobacteria* (55.6% versus 41.9%, respectively).

10.1128/mBio.00590-17.10 TABLE S5  Core bacterial community (CBC) OTUs for SA, SB, and D, as well as shared OTUs. Download TABLE S5, DOCX file, 0.05 MB. Copyright © 2017 Svensson et al. 2017 Svensson et al. This content is distributed under the terms of the Creative Commons Attribution 4.0 International license.

Next, the CBCs of clusters SA, SB, and D were compared based on the percentage of CBC OTUs belonging to different phyla (Fig. 4A, right panel). This revealed that CBC-SA, with the majority of reads from *Betaproteobacteria* (53.8%), actually had a similar proportion of OTUs from this group (21/85 CBC OTUs [24.7%]) as from *Alphaproteobacteria* (18/85 [21.1%]) or *Acidobacteria* (17/85 [20%]). Likewise, CBC-SB, while having more than 90% of reads from *Betaproteobacteria*, had a comparatively large proportion of *Firmicutes* (*Clostridiales* accounted for 5/26, or 19.2%, of OTUs). Finally, CBC-D had more *Gammaproteobacteria* species (9/18 OTUs) compared to the CBCs of SA and SB. Definition of the core bacterial species of each KC community therefore suggested that the *Betaproteobacteria* dominated all three communities but with significant differences between the constituent families of species.

We next identified shared or unique species between the three CBCs (Table S5). Only three OTUs were shared by all three CBCs (one *Oxalobacteraceae* and two pseudomonads). Both taxa include common soil bacteria with diverse metabolic capabilities (29, 30). CBC-SA and -SB (both shallow/low pH) shared more OTUs with each other (10 OTUs) than either one shared with cluster D (5 OTUs each [Fig. 4B]). Three of the OTUs shared by only the CBCs of SA and SB (but absent from CBC-D) were from *Alphaproteobacteria* lineages, including nitrogen-fixing *Rhizobiales* normally associated with plant roots, which suggested that the shallower communities may be influenced by the rhizosphere. A notable difference between CBC-SA and CBC-SB was the number of *Acidobacteria* OTUs (20% versus 4%, respectively). In contrast, we observed more *Clostridiales* OTUs in CBC-SB (20% versus 3.5% for CBC-SA). The five *Clostridiales* OTUs of CBC-SB included two potential *Desulfosporosinus* species and one *Thermosinus* species, both of which include sulfate-reducing bacteria (31–33). For the CBC of cluster D, there was greater representation from *Gammaproteobacteria* (such as xanthomonads and pseudomonads), *Acinetobacter*, and *Serratia*. Also uniquely present in CBC-D was a member of the *Anaerolineae*, a *Chloroflexi* whose cultivated members appear to be strictly anaerobic (34, 35).

### Iron bacteria mark the KC microbiome near the surface.

Acid-soluble metal ions, such as iron species, are important for the activity of other antibacterial clays (13), and may be responsible for the activity of KC (S. Behroozian and J. Davies, unpublished data), which is relatively rich in iron (15, 22). To gain insight into the ferruginous environment of KC, we focused on clay bacteria that may play roles in iron cycling. Interestingly, many iron bacteria (those that perform dissimilatory iron oxidization at near neutral pH) that have been characterized are *Betaproteobacteria* (36, 37), which were dominant in KC. Numerous comamonads (142 OTUs), which may include iron-oxidizing *Leptothrix* spp., were identified in the total sequence data set, and several were defined as part of CBC-SA, CBC-SB, and CBC-D (Table S5). We also identified genera of iron bacteria or bacteria that can tolerate heavy metals such as *Delftia* (38) and *Acidivorax* (36).

**FIG 5:**
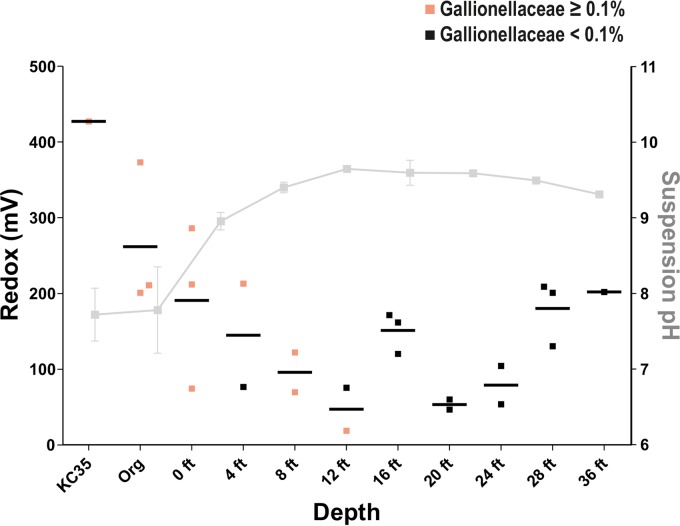
Prevalence of iron-oxidizing bacteria in KC compared to depth, pH, and redox. Redox measured for KC core samples (left *y* axis) versus depth (*x* axis) is shown. Communities with ≥0.1% reads mapping to the order *Gallionellaceae* are shown. The mean pH of each depth is shown in gray.

Notably, a *Gallionellaceae* OTU was shared only by CBC-SA and CBC-SB but was not present in the CBC we defined for cluster D. The type species of this family (*Gallionella ferruginea*) colonizes the transition zone between anaerobic and aerobic environments (redox potential of +200 to +320 mV and low oxygen tensions of 0.1 to 1 mg/liter) and participates in dissimilatory iron oxidation, requiring Fe(II) and oxygen for growth (39; reviewed in references 36 and 37). For KC samples, we observed a trend of decreasing redox potential from 0 to 12 ft (approximately +300 to +50 mV) and an increase in redox from 20 to 36 ft (+50 to +200 mV) (Fig. 5). Interior samples tended to have a lower potential (+50 to +150 mV). Samples with at least 0.1% of reads from *Gallionellaceae* OTUs (all 14 identified in our studies) were from 12 ft and above. The absence of *Gallionella* below 12 ft is consistent with redox measurements of these samples, which were below +200 mV and the previously reported redox niche of these bacteria (39). When the pH of each clay sample was included, all samples with *Gallionella* had circumneutral, or even slightly acidic pH (Fig. 5). In addition, all samples that had at least 0.1% of reads as *Gallionellaceae* species were part of clusters SA and SB, except for Kis1-12 (Fig. S2).

10.1128/mBio.00590-17.3 FIG S2  Presence of *Gallionellaceae* sequences in KC sample amplicons, shown according to PCoA clusters (SA, SB, and D). Download FIG S2, PDF file, 0.2 MB. Copyright © 2017 Svensson et al. 2017 Svensson et al. This content is distributed under the terms of the Creative Commons Attribution 4.0 International license.

### Antibacterial and economic potential of KC.

**FIG 6:**
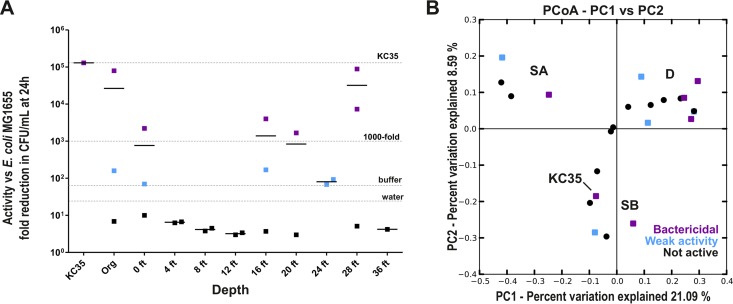
Antibacterial activity of KC core samples. Suspensions (10 mg/ml) were prepared from dried clay in water from each core sample and tested for antibacterial activity against *E. coli* MG1655. Bacteria in log phase were added to suspensions and incubated at 37°C with shaking. After 24 h, samples were serially diluted and plated for CFU on LB agar. (A) Higher antibacterial activity is detected at the edges of the deposit. The mean fold reduction (*n* = 4) for each depth (short lines) and the value for each individual sample (symbols) are plotted. Organic samples and KC35 are plotted separately. Samples with weak activity (blue squares) were defined as those resulting in at least a 66-fold decrease in viable CFU after 24 h, the reduction observed for phosphate buffer (pH 4.3). “Bactericidal” samples (Purple squares) were defined as those reducing viable CFU by at least 1,000-fold. For comparison, the activity of KC35 and water alone are indicated. (B) KC community structure is not predictive of clay activity. The results of unweighted UniFrac PCoA analysis of core sample communities with respect to activity against *E*. *coli* MG1655 are indicated as follows: weak activity (>66-fold reduction in CFU); bactericidal (>1,000-fold reduction); not active (less activity than phosphate buffer).

KC exhibits potent inhibitory activity against a variety of bacteria and other microbes, including multidrug-resistant ESKAPE pathogens (20) and pathogenic fungi (Behroozian et al., unpublished). To gain insight into what might contribute to the antibacterial activity of KC and how this activity might shape its resident communities, we also determined the antibacterial activity of each sample used for microbiome analysis against *E. coli* MG1655. The activity of KC core samples was highly variable (Fig. 6A). While KC35 caused an approximately 5-log-unit decrease in CFU during incubation, the KC core samples varied from bactericidal (purple squares show greater than 3-log-unit killing) to more weakly active (blue squares show less than 1,000-fold decrease in CFU, but higher than water alone) (Fig. 6A). Some samples actually enhanced viability of the test inoculum compared to the H_2_O control (black squares) (Fig. 6A). While we did not identify a correlation between community type and activity, even though depth appeared to affect microbiome composition, all samples from the interior (i.e., 4 to 12 ft) showed little or no activity. There was also no correlation between activity and core (i.e., Kis3 versus Kis1 [not shown]). Finally, we sought to determine whether activity correlated with the type of bacterial community type identified by PCoA analysis (Fig. 2B and C), as we hypothesized, for example, that communities marking a particular redox environment could affect the speciation of antibacterial Fe ions. However, there was an almost random distribution of high-activity samples (Fig. 6B). There was a very weak correlation between redox potential and antibacterial activity, with samples with higher redox potential tending to show greater reduction of CFU (Fig. S3), while the presence of *Gallionella* showed no correlation with activity.

10.1128/mBio.00590-17.4 FIG S3  Redox potential, antibacterial activity, and *Gallionella* sequences. Download FIG S3, PDF file, 0.1 MB. Copyright © 2017 Svensson et al. 2017 Svensson et al. This content is distributed under the terms of the Creative Commons Attribution 4.0 International license.

Soils are rich sources of economically valuable bacterial species, such as those that produce novel antimicrobials or with plant growth-promoting activities. Up to 3% of reads for KC samples were assigned to the *Actinomycetales* (Fig. S4A). This group includes many well-known producers of bioactive and antimicrobial secondary metabolites (40). Several species of *Paenibacillus*, a genus that includes nitrogen fixers with plant growth-promoting ability such as *Paenibacillus polymyxa* (41), are also present in KC (Fig. S4A). Preliminary culturing studies isolated viable bacteria on Actinomycete-selective agar at relatively high CFU (Fig. S4B).

10.1128/mBio.00590-17.5 FIG S4  Potentially economically or medically interesting bacteria in Kis3 samples. Download FIG S4, PDF file, 0.1 MB. Copyright © 2017 Svensson et al. 2017 Svensson et al. This content is distributed under the terms of the Creative Commons Attribution 4.0 International license.

## DISCUSSION

Characterizing resident microbial populations is essential for a complete understanding of terrestrial environments and systems and identification of microbes with economically useful capabilities. Here, we have characterized the bacteria that colonize the Kisameet Bay glacial clay deposit, which is the source of potently antibacterial KC. The deposit harbors surprising bacterial diversity, with greater than 300 species in most samples and thousands of species in samples mixed with the organic overburden. In fact, our estimates may be an underestimate due to sequencing depth. Previous analyses of the bacterial diversity present in Boom clay borehole water found that this environment contained only 100 to 150 OTUs (42). Diversity in the Boom clay microbiome correlated weakly with total organic carbon measures (42). While we did not measure organic carbon in this study, the organic overburden is likely responsible for the increase in diversity in some samples from the deposit. Soil pH is also a strong predictor of bacterial diversity, with neutral environments harboring more diversity and species richness than environments with either low or high pH (43). The high pH of the interior of the KC deposit is consistent with its slightly lower bacterial diversity. In iron-rich freshwater wetlands, bacteria such as *Gallionella* tend to be more abundant in the spring compared to summer or fall (44, 45). Sampling of KC in different seasons may reveal variable temporal bacterial community dynamics. Profiling of resident *Archaea*, *Eukarya*, or bacteriophages will also further an understanding of the microbial diversity of KC.

To date, there have been few studies of the bacterial content of native clays. Bacteria enriched from a desiccated sample of commercially available Wyoming bentonite clay were found to be closely related to *Desulfovibrio africanus*, a sulfate-reducing deltaproteobacterium (46). We did not detect this species in our survey; however, the deeper samples of cluster D did share a deltaproteobacterium OTU. Two studies have addressed the microbiome of the deep subsurface Boom clay. A 16S rRNA clone library approach tentatively identified 11 different OTUs in the clay (47). Interestingly, *Proteobacteria* made up 76% of the species, with *Betaproteobacteria*, such as the comamonad *Acidovorax*, accounted for 46%. Our survey of KC bacteria likewise suggests a dominance of *Betaproteobacteria*. In addition, both the CBCs defined in our survey and the species identified in the Boom clay revealed *Gammaproteobacteria* such as *Pseudomonas* and *Acinetobacter*.

We note that KC harbors at least three major bacterial community types. Two different communities (SA and SB) were identified in more shallow/lower pH samples. Comparison of the CBCs of these clusters showed that cluster SA had more *Acidobacteria* (27, 48, 49) than cluster SB. In contrast, acidobacteria were absent from the CBC defined for the deeper cluster D. The *Acidobacteria* is a relatively new, poorly characterized, and diverse phylum, although members of this group have been identified in many different environments, where they may constitute up to 20% of bacteria (27, 49, 50). We note that the two samples composed of mostly organic material were part of cluster SA. Cluster SA may represent communities in soil like KC under higher organic material or oxygen tension. Acidobacteria were not reported in the Boom clay (42, 47) but were found to be relatively abundant in 16S rRNA libraries prepared from low-pH, clay-rich Cerrado soil (51). The presence of this clade in CBC-SA may therefore reflect a more soil-like, rather than clay-like, environment. Certainly, the correlation between the horizontal location of the core and the PCoA cluster for shallow samples (i.e., the tendency of surface samples from cores extracted closer to the edge of the deposit, such as Kis1, Kis3, and Kis5, to be part of cluster SA) provides an impetus to explore correlations of these communities with their macroflora neighbors.

In contrast to CBC-SA, CBC-SB included four potential sulfate-reducing clostridia—three *Desulfosporosinus* species and a *Thermosinus* species. Boom clay likewise included numerous clostridia such as sulfate-reducing *Desulfotomaculum* (42). Sulfate-reducing bacteria participate in anaerobic dissimilatory reduction of sulfate to compounds such as hydrogen sulfide, and are often associated with decaying organic matter (52). Thus, cluster SB may reveal environments that are more anaerobic, while still in proximity to the overburden layer of the deposit. Interestingly, cyclic S_8_ extracted from KC with organic solvents (J. Tan and J. Davies, unpublished observations), has antibacterial activity (53–55). Therefore, microbial sulfur cycling may contribute to the complexity of KC. Furthermore, *Desulfosporosinus* and *Thermosinus* strains have been identified that can reduce not only sulfate but also Fe(III) (56, 57), supporting the presence of both these *Clostridia* and iron bacteria in KC.

KC is iron-rich (15, 22), and potential iron bacteria were identified in our survey. In particular, *Gallionella*-like iron bacteria are found in shallower samples of KC. *Gallionella* spp. are microaerobic iron oxidizers that live at iron-rich oxic/anoxic interfaces (36). While they have been described as inhabiting neutral pH zones, acid-tolerant species of *Gallionella* have been identified, and *Gallionella*-like organisms are increasingly found in heavy metal-rich acid mine drainage (58–64). Bulk clay iron levels are relatively uniform across the deposit (data not shown), but the low pH of some regions may affect the solubility and availability of iron species. KC provides an interesting environment for studying iron bacteria, which are associated with biofouling and the deterioration of pipes and water distribution systems (36), and the 14 *Gallionellaceae* OTUs identified in KC may represent species with novel characteristics. Iron-oxidizing bacteria have been shown to have potential bioagents removing heavy metals (65).

What makes KC antibacterial? Previous characterization of density-separated KC fractions showed that antibacterial activity was higher in those fractions that had higher water-leachable metal content (W. Xu, S. Behroozian, W. Yenjaichon, J. Grace, J. Davies, and L. Li, unpublished data). Low pH also affects KC activity, which may contribute to the solubility of ions such as Fe(II), Fe(III), or Al(III) (Behroozian et al., unpublished). We found a weak relationship between redox condition, which might affect the speciation or solubility of antibacterial ions, such as iron, and antibacterial activity. Alternatively, specific redox regions may indicate regions of the clay where antibacterial activity resides. For example, Jordan’s red clay harbors bacteria of the genus *Lysobacter*, xanthomonads that are important biocontrol agents and sources of exoenzymes and antibiotics (66), which appear to be responsible for its antibacterial activity (19). However, we found no strong correlation between bacterial community type and activity of the clay. Highly active KC35 had an increased number of *Gallionella* reads, but we found no correlation between *Gallionella* and activity in core samples. Acidobacteria are known to produce antibiotics such as polyketides (67). KC awaits more-focused culturing studies for the identification of useful bacteria. While we cannot rule out the possibility that particular bacterial species contribute to its inhibitory properties, the bioactivity of KC does not strongly shape community structure.

KC is a very complex natural material, and many different factors may contribute to its unique properties, including the resident bacterial communities. If clays and other natural products are to be used as therapeutic agents, their resident bacteria must be characterized, as they may alter clay structure and affect the efficacy and/or consistency of preparations. KC bacteria should be examined in more detail for the production of novel bioactive compounds or useful metabolic capabilities. In addition, future microbiome studies of natural environments, such as the KC deposit with the unique chemical, absorptive, diffusion, and microbial properties of KC, may uncover novel microbial interactions involving small bioactive molecules, antimicrobial metal ions, nutrients, and oxygen availability. The KC deposit may serve as an environment in which to observe novel chemical and genetic interactions between microbes, to study ecological succession, and to reveal new bacterial types with valuable economic properties.

## MATERIALS AND METHODS

### Harvesting and storage of core samples.

Five vertical cores (Kis1 to Kis5) were harvested from the Kisameet Bay deposit (51°58′16″ N/127°52′56″ W) on 24 October 2012. Following transport to the University of British Columbia, cores were stored sealed at 4°C under normal atmospheric conditions in the dark. The cores were opened, and the top 10 cm from each 4 ft length of core was saved for analyses of physical, chemical, and biotic characteristics. To avoid cross-contamination between depths, only the interior undisturbed part of each core was used for analysis. To measure antibiotic activity, a portion of each core sample was dried in a desiccator at room temperature under vacuum for 2 to 4 days, ground to a fine powder with a mortar and pestle, autoclaved for 20 min, and stored at room temperature in the dark prior to testing. For metagenome analysis and culturing studies, unautoclaved, native clay was used. The sample activity, physicochemical characteristics, and bacterial community were compared to that of the previously characterized, highly active sample KC35 (20).

### Measurement of KC mineralogical and physicochemical characteristics.

Details of the analysis of the abiotic properties of KC core samples (mineralogy by X-ray diffraction, pH, redox, and elemental analyses by inductively coupled plasma optical emission spectroscopy) are presented in Text S1 in the supplemental material.

10.1128/mBio.00590-17.1 TEXT S1 Supplemental methods. Download TEXT S1, DOCX file, 0.04 MB. Copyright © 2017 Svensson et al. 2017 Svensson et al. This content is distributed under the terms of the Creative Commons Attribution 4.0 International license.

### Extraction of DNA from KC.

Total DNA was extracted from wet, unautoclaved clay from each core sample using the FastDNA kit for soil (MP Biomedicals, Santa Ana, CA) according to the manufacturer’s instructions with minor modifications. Briefly, ~400 mg of wet, unautoclaved clay was added to a tube with ~500 mg of silica beads (1-mm diameter; Sigma). Samples were shaken on a FastPrep homogenizer (MP Biomedicals) for 40 s at a speed of 6.0. Following removal of particulate matter at 14,000 × *g* for 10 min, the clarified extract was applied to a spin column and purified according to the manufacturer’s recommendations. DNA was eluted with water and quantified using a Nanodrop spectrophotometer.

### Next-generation sequencing of KC 16S rRNA amplicons and sequence data analysis.

Sequencing of the 16S rRNA gene variable 3 (V3) region, amplified by PCR with primers 341F (F stands for forward) and 518R (R stands for reverse) from total DNA extracted from KC core samples, was performed as previously described (68, 69) on an Illumina Miseq platform. Details of library preparation and sequencing can be found in Text S1. Details of data quality control and analysis using Qiime (26) are likewise outlined in detail in Text S1.

### Isolation of KC-resident bacteria.

Viable bacteria from KC core samples and KC35 were cultured from unautoclaved clay. To determine viable counts of *Actinomycete*-like bacteria, equal weights of wet unautoclaved clay from each core sample that had been stored under native atmospheric conditions at 4°C (approximately 100 mg) were suspended in 10-ml portions of sterile saline, vortexed, and serially diluted in sterile saline. Dilutions were plated on *Actinomycete* isolation agar (AIA) and incubated at 30°C for 4 to 6 days until colonies were visible.

### Antibacterial activity of core samples.

Quantification of antibacterial activity was performed essentially as previously described (20) using the wild-type *Escherichia coli* K-12 strain MG1655 (70), grown aerobically at 37°C in Luria-Bertani broth (LB). Each clay sample was tested at a concentration of 10 mg/ml, as described in Text S1.
