# Supplementary material for: Kisameet Glacial Clay: an Unexpected Source of Bacterial Diversity
Source: mBio. 2017 May 23;8(3):e00590-17. doi: 10.1128/mBio.00590-17 (PMC5442455; doi:10.1128/mBio.00590-17)
Supplement: TABLE S1 [file mbo003173310st1.docx]

**Table S1.** **Library and read statistics for next generation sequencing.** OTUs with only one or two reads (singletons or doubletons) were removed prior to analyses.

| **Num samples:** 24 | **Sample ID** | **Description** | **Seqs /**  **sample** | **Visible organic matter** | **Total OTUs** | **Cluster (PCoA)** |
| --- | --- | --- | --- | --- | --- | --- |
| **Total OTUs:** 5,032 | SS1 | KC35 | 137,279 | no | 458 | SB |
| **Num observations (sequences):** 4,907,848 | SS2 | Kis3-0ft | 198,486 | no | 2,197 | SA |
|  | SS3 | Kis1-0ft | 333,878 | no | 827 | D |
| **Seqs / sample summary:** | SS4 | Kis1-28ft | 264,398 | no | 281 | D |
| **Min:** 46,471 | SS5 | Kis3-4ft | 207,315 | no | 685 | D |
| **Max:** 333,878 | SS6 | Kis3-8ft | 133,820 | no | 1,439 | SA |
| **Median:** 206,435 | SS7 | Kis3-12ft | 121,535 | no | 921 | D |
| **Mean:** 196,313.92 | SS8 | Kis3-16ft | 146,153 | no | 659 | D |
| **Std. dev.:** 80,627.16 | SS9 | Kis3-20ft | 46,471 | no | 313 | D |
| **Median Absolute Deviation:** 64,305.08 | SS10 | Kis3-24ft | 263,027 | no | 786 | D |
|  | SS11 | Kis3-28ft | 78,019 | no | 360 | D |
|  | SS12 | Kis2-0ft | 306,856 | no | 632 | SB |
|  | SS13 | Kis4-0ft | 229,954 | no | 978 | SB |
|  | SS14 | Kis5-0ft | 296,000 | yes | 1,463 | SA |
|  | SS15 | Kis2-36ft | 327,196 | no | 684 | D |
|  | SS16 | Kis4-16ft | 206,435 | no | 462 | D |
|  | SS17 | Kis5-28ft | 244,504 | no | 582 | D |
|  | SS18 | Kis1-4ft | 241,295 | no | 794 | SB |
|  | SS19 | Kis1-8ft | 213,933 | no | 1,176 | SB |
|  | SS20 | Kis1-12ft | 226,164 | no | 1,145 | D |
|  | SS21 | Kis1-16ft | 160,305 | no | 459 | D |
|  | SS22 | Kis1-20ft | 183,524 | no | 444 | D |
|  | SS23 | Kis1-24ft | 178,458 | no | 553 | D |
|  | SS25 | Kis3-0ft org | 110,483 | yes | 2,410 | SB |
