# Supplementary material for: Kisameet Glacial Clay: an Unexpected Source of Bacterial Diversity
Source: mBio. 2017 May 23;8(3):e00590-17. doi: 10.1128/mBio.00590-17 (PMC5442455; doi:10.1128/mBio.00590-17)
Supplement: TABLE S2 [file mbo003173310st2.docx]

**Table S2.** Mineralogical composition of selected KC core samples. The percent of each indicated mineral was determined by XRD.

| **Mineral** | **Ideal Formula** | **KC35** | **1-0*** | **1-12** | **1-24** | **2-0*** | **2-36** | **3-0*** | **3-12** | **3-28** | **4-0*** | **4-16** | **5-0** | **5-28** |
| --- | --- | --- | --- | --- | --- | --- | --- | --- | --- | --- | --- | --- | --- | --- |
| **Actinolite** | Ca_2_(Mg,Fe^2+^)_5_Si_8_O_22_(OH)_2_ | 9.3 | 9.9 | 10.8 | 10.0 | 10.1 | 9.9 | 16.3 | 9.5 | 8.9 | 10.6 | 10.9 | 10.1 | 9.8 |
| **Albite low** | NaAlSi_3_O_8_ | 40.8 | 39.0 | 37.7 | 37.8 | 38.6 | 43.3 | 39.6 | 36.2 | 44.7 | 40.3 | 38.4 | nd | nd |
| **Albite low, calcian** | (Na,Ca)(AlSi)_4_O_8_ | nd** | nd | nd | nd | nd | nd | nd | nd | nd | nd | nd | 37.3 | 38.3 |
| **Biotite 1M** | K(Mg,Fe)_3_AlSi_3_O_10_(OH)_2_ | 10.0 | 11.5 | 13.6 | 14.4 | 11.7 | 5.9 | 8.8 | 15.5 | 3.3 | 10.6 | 14.0 | 13.9 | 14.5 |
| **Calcite** | CaCO_3_ | nd | nd | nd | nd | nd | nd | nd | nd | nd | nd | nd | 0.3 | nd |
| **Clinochlore II** | (Mg,Fe^2+^)_5_Al(Si_3_Al)O_10_(OH)_8_ | 9.0 | 10.3 | 11.4 | 12.7 | 11.0 | 7.6 | 9.4 | 13.3 | 5.2 | 8.5 | 9.7 | 8.7 | 11.6 |
| **Goethite** | FeO(OH) | nd | nd | nd | nd | nd | nd | nd | nd | nd | nd | nd | nd | nd |
| **Gypsum** | CaSO_4_·2H_2_O | 0.9 | 0.7 | nd | nd | nd | 0.3 | nd | 0.2 | nd | nd | nd | nd | nd |
| **Illite-Muscovite 2M1** | K_0.65_Al_2.0_Al_0.65_Si_3.35_O_10_(OH)_2_ | 5.3 | 5.2 | 5.5 | 5.8 | 5.4 | 4.6 | 4.5 | 5.2 | 2.3 | 5.2 | 5.7 | 8.3 | 5.9 |
| **Laumontite** | CaAl_2_Si_4_O_12_·4H_2_O | 3.5 | 4.1 | 3.5 | 3.1 | 4.1 | 1.9 | 3.0 | 4.4 | 2.0 | 3.8 | 3.4 | 3.8 | 3.4 |
| **Magnesite** | MgCO_3_ | nd | nd | nd | nd | nd | nd | nd | nd | nd | nd | nd | nd | nd |
| **Magnetite** | Fe_3_O_4_ | nd | nd | nd | 1.1 | nd | nd | nd | nd | 1.1 | nd | nd | nd | nd |
| **Microcline ordered** | KAlSi_3_O_8_ | 8.9 | 8.7 | 9.1 | 8.6 | 9.0 | 8.9 | 8.2 | 8.8 | 10.0 | 9.5 | 9.4 | 8.5 | 8.9 |
| **Quartz low** | SiO_2_ | 12.2 | 10.5 | 8.5 | 7.7 | 10.0 | 17.5 | 10.2 | 6.9 | 22.5 | 11.5 | 8.5 | 9.1 | 7.6 |
| **Total** |  | 100 | 100 | 100 | 100 | 100 | 100 | 100 | 100 | 100 | 100 | 100 | 100 | 100 |

*0 – 4 ft blend-bulk sample

**nd – not determined
