# Supplementary material for: Kisameet Glacial Clay: an Unexpected Source of Bacterial Diversity
Source: mBio. 2017 May 23;8(3):e00590-17. doi: 10.1128/mBio.00590-17 (PMC5442455; doi:10.1128/mBio.00590-17)
Supplement: TABLE S3 [file mbo003173310st3.docx]

**Table S3.** Acid-digested elemental composition of KC core sample bulk clay. Analyte levels were determined by ICP-OES and are reported in mg/kg. MDM – minimum detection limit (mg/kg).

| **Analyte** (emission, nm) | **MDM** | **KC35** | **1-0** | **1-4** | **1-8** | **1-12** | **1-16** | **1-20** | **1-24** | **1-28** | **2-0** | **2-36** | **3-0** | **3-4** | **3-8** | **3-12** | **3-16** | **3-20** | **3-24** | **3-28** | **4-0** | **4-16** | **5-0** | **5-28** |
| --- | --- | --- | --- | --- | --- | --- | --- | --- | --- | --- | --- | --- | --- | --- | --- | --- | --- | --- | --- | --- | --- | --- | --- | --- |
| **Ag** (328.068) | 0.0024 | 0.00E+0 | 0.00E+0 | 0.00E+0 | 0.00E+0 | 0.00E+0 | 0.00E+0 | 0.00E+0 | 0.00E+0 | 0.00E+0 | 0.00E+0 | 0.00E+0 | 0.00E+0 | 0.00E+0 | 0.00E+0 | 0.00E+0 | 0.00E+0 | 0.00E+0 | 0.00E+0 | 0.00E+0 | 0.00E+0 | 0.00E+0 | 0.00E+0 | 0.00E+0 |
| **Al** (396.153) | 0.0083 | 4.70E+4 | 4.25E+4 | 3.68E+4 | 2.91E+4 | 2.80E+4 | 3.72E+4 | 3.18E+4 | 3.34E+4 | 3.35E+4 | 2.51E+4 | 3.20E+4 | 3.46E+3 | 3.63E+4 | 3.58E+4 | 3.30E+4 | 3.34E+4 | 3.48E+4 | 3.33E+4 | 2.94E+4 | 1.52E+4 | 3.36E+4 | 8.16E+2 | 3.46E+4 |
| **As** (188.979) | 0.0361 | 0.00E+0 | 0.00E+0 | 0.00E+0 | 0.00E+0 | 0.00E+0 | 0.00E+0 | 0.00E+0 | 0.00E+0 | 0.00E+0 | 0.00E+0 | 0.00E+0 | 0.00E+0 | 0.00E+0 | 0.00E+0 | 0.00E+0 | 0.00E+0 | 0.00E+0 | 0.00E+0 | 0.00E+0 | 0.00E+0 | 0.00E+0 | 0.00E+0 | 0.00E+0 |
| **Ba** (233.527) | 0.0006 | 5.61E+2 | 4.90E+2 | 4.52E+2 | 3.67E+2 | 3.88E+2 | 4.78E+2 | 4.17E+2 | 4.42E+2 | 3.89E+2 | 2.84E+2 | 3.36E+2 | 1.39E+1 | 4.27E+2 | 4.83E+2 | 4.44E+2 | 4.47E+2 | 4.59E+2 | 4.47E+2 | 2.55E+2 | 1.74E+2 | 4.34E+2 | 1.00E+1 | 4.44E+2 |
| **Be** (313.107) | 0.0005 | 3.41E+0 | 3.11E+0 | 2.87E+0 | 1.99E+0 | 2.06E+0 | 2.35E+0 | 2.16E+0 | 2.29E+0 | 2.31E+0 | 1.87E+0 | 1.98E+0 | 0.00E+0 | 2.81E+0 | 2.37E+0 | 2.65E+0 | 2.74E+0 | 2.70E+0 | 2.46E+0 | 1.99E+0 | 1.24E+0 | 2.37E+0 | 0.00E+0 | 2.42E+0 |
| **Ca** (317.933) | 0.0100 | 1.42E+4 | 1.25E+4 | 1.14E+4 | 7.53E+3 | 6.79E+3 | 8.47E+3 | 6.89E+3 | 7.99E+3 | 9.99E+3 | 6.77E+3 | 1.33E+4 | 1.27E+3 | 1.13E+4 | 1.14E+4 | 1.08E+4 | 1.12E+4 | 1.10E+4 | 1.10E+4 | 1.70E+4 | 4.38E+3 | 7.82E+3 | 1.00E+3 | 9.51E+3 |
| **Cd** (228.802) | 0.0019 | 1.92E+0 | 3.16E+0 | 2.26E+0 | 0.00E+0 | 2.66E+0 | 0.00E+0 | 0.00E+0 | 5.51E-1 | 2.81E+0 | 0.00E+0 | 2.98E+0 | 6.01E+0 | 2.45E+0 | 0.00E+0 | 0.00E+0 | 1.71E+0 | 0.00E+0 | 0.00E+0 | 4.66E+0 | 2.53E+0 | 1.39E+0 | 3.13E+0 | 1.40E+0 |
| **Co** (228.616) | 0.0026 | 4.02E+1 | 3.54E+1 | 3.42E+1 | 2.45E+1 | 2.56E+1 | 3.05E+1 | 2.69E+1 | 2.88E+1 | 2.87E+1 | 2.38E+1 | 3.01E+1 | 1.32E+0 | 3.34E+1 | 3.31E+1 | 3.06E+1 | 3.22E+1 | 5.85E+2 | 2.93E+1 | 2.81E+1 | 1.47E+1 | 2.89E+1 | 1.00E+0 | 2.92E+1 |
| **Cr** (267.716) | 0.0011 | 3.37E+1 | 2.89E+1 | 3.00E+1 | 2.11E+1 | 2.35E+1 | 2.21E+1 | 2.05E+1 | 2.57E+1 | 2.41E+1 | 2.06E+1 | 4.04E+1 | 1.26E+1 | 2.90E+1 | 2.68E+1 | 2.92E+1 | 3.19E+1 | 2.60E+1 | 2.47E+1 | 4.54E+1 | 1.98E+1 | 2.38E+1 | 4.81E+0 | 2.28E+1 |
| **Cu** (327.393) | 0.0042 | 1.76E+2 | 1.08E+2 | 7.40E+2 | 1.21E+2 | 1.02E+2 | 8.00E+1 | 8.16E+1 | 8.31E+1 | 1.11E+2 | 1.11E+2 | 1.83E+2 | 1.17E+2 | 1.13E+2 | 1.37E+2 | 2.15E+2 | 1.77E+2 | 2.86E+2 | 1.13E+2 | 2.49E+2 | 1.43E+2 | 8.12E+1 | 6.24E+1 | 2.82E+2 |
| **Fe** (238.204) | 0.0077 | 3.32E+4 | 2.02E+4 | 4.32E+4 | 1.11E+4 | 2.81E+4 | 9.36E+3 | 1.45E+4 | 1.56E+4 | 1.94E+4 | 2.84E+4 | 3.23E+4 | 1.33E+3 | 2.32E+4 | 3.86E+4 | 4.89E+4 | 4.79E+4 | 3.41E+4 | 2.89E+4 | 4.05E+4 | 3.50E+4 | 1.66E+4 | 9.13E+2 | 1.64E+4 |
| **K** (766.490) | 0.0500 | 1.83E+4 | 1.57E+4 | 1.56E+4 | 1.14E+4 | 1.31E+4 | 1.39E+4 | 1.33E+4 | 1.42E+4 | 1.38E+4 | 9.81E+3 | 1.17E+4 | 5.99E+2 | 1.45E+4 | 1.63E+4 | 1.52E+4 | 1.51E+4 | 1.51E+4 | 1.41E+4 | 9.27E+3 | 6.08E+3 | 1.41E+4 | 3.17E+2 | 1.43E+4 |
| **Mg** (285.213) | 0.6521 | 2.88E+4 | 2.51E+4 | 2.45E+4 | 1.70E+4 | 1.89E+4 | 2.03E+4 | 1.95E+4 | 2.05E+4 | 2.10E+4 | 1.56E+4 | 2.22E+4 | 5.63E+2 | 2.22E+4 | 2.38E+4 | 2.28E+4 | 2.22E+4 | 2.22E+4 | 2.14E+4 | 2.00E+4 | 9.47E+3 | 2.09E+4 | 3.98E+2 | 2.10E+4 |
| **Mn** (257.610) | 0.0136 | 1.47E+3 | 1.28E+3 | 1.22E+3 | 9.49E+2 | 9.99E+2 | 1.20E+3 | 1.05E+3 | 1.09E+3 | 1.09E+3 | 7.66E+2 | 1.10E+3 | 2.36E+1 | 1.13E+3 | 1.22E+3 | 1.16E+3 | 1.16E+3 | 1.15E+3 | 1.11E+3 | 9.52E+2 | 4.63E+2 | 1.09E+3 | 1.72E+1 | 1.10E+3 |
| **Mo** (202.031) | 0.0058 | 1.31E+0 | 6.25E-1 | 1.44E+0 | 0.00E+0 | 0.00E+0 | 0.00E+0 | 0.00E+0 | 2.78E-1 | 3.43E-1 | 2.06E+0 | 6.02E+0 | 1.69E+0 | 1.11E+0 | 0.00E+0 | 1.67E+0 | 0.00E+0 | 3.61E+0 | 0.00E+0 | 2.25E+0 | 2.45E+0 | 0.00E+0 | 1.44E+0 | 0.00E+0 |
| **Na** (589.592) | 0.0500 | 1.40E+3 | 1.12E+3 | 1.27E+3 | 1.38E+3 | 1.86E+3 | 2.28E+3 | 1.71E+3 | 1.35E+3 | 1.12E+3 | 8.74E+2 | 9.90E+2 | 0.00E+0 | 1.20E+3 | 1.04E+3 | 1.11E+3 | 9.13E+2 | 1.23E+3 | 1.28E+3 | 1.18E+3 | 5.14E+2 | 1.98E+3 | 0.00E+0 | 1.59E+3 |
| **Ni** (231.604) | 0.0040 | 2.96E+1 | 2.49E+1 | 2.65E+1 | 1.83E+1 | 1.91E+1 | 1.99E+1 | 1.83E+1 | 2.20E+1 | 2.35E+1 | 1.96E+1 | 3.50E+1 | 6.39E+0 | 2.54E+1 | 2.39E+1 | 2.37E+1 | 3.89E+1 | 7.75E+2 | 2.19E+1 | 4.06E+1 | 1.63E+1 | 2.07E+1 | 3.56E+0 | 2.72E+1 |
| **Pb** (220.353) | 0.0029 | 3.19E+1 | 2.31E+1 | 5.05E+1 | 2.04E+1 | 1.71E+1 | 8.75E+0 | 9.87E+0 | 1.04E+1 | 1.48E+1 | 1.69E+1 | 3.56E+1 | 3.53E+1 | 4.10E+1 | 2.11E+1 | 2.15E+1 | 3.54E+1 | 2.21E+1 | 1.70E+1 | 3.51E+1 | 2.48E+1 | 4.65E+1 | 1.25E+1 | 1.29E+1 |
| **Sb** (206.836) | 0.0204 | 8.63E+0 | 7.76E+0 | 6.01E+0 | 5.68E+0 | 5.10E+0 | 5.76E+0 | 6.30E+0 | 4.39E+0 | 5.04E+0 | 5.03E+0 | 8.67E+0 | 3.21E+0 | 4.60E+0 | 8.39E+0 | 6.89E+0 | 9.04E+0 | 5.98E+0 | 7.11E+0 | 1.11E+1 | 6.38E+0 | 7.45E+0 | 2.69E+0 | 5.01E+0 |
| **Se** (196.026) | 0.0375 | 0.00E+0 | 0.00E+0 | 0.00E+0 | 0.00E+0 | 0.00E+0 | 0.00E+0 | 0.00E+0 | 0.00E+0 | 0.00E+0 | 0.00E+0 | 0.00E+0 | 0.00E+0 | 0.00E+0 | 0.00E+0 | 0.00E+0 | 0.00E+0 | 0.00E+0 | 0.00E+0 | 0.00E+0 | 0.00E+0 | 0.00E+0 | 2.70E+0 | 0.00E+0 |
| **Sn** (189.927) | 0.0106 | 2.00E+1 | 2.41E+1 | 2.11E+1 | 1.16E+1 | 1.48E+1 | 1.41E+1 | 1.24E+1 | 1.48E+1 | 1.47E+1 | 1.28E+1 | 2.16E+1 | 1.09E+1 | 2.23E+1 | 2.12E+1 | 1.96E+1 | 2.71E+1 | 1.79E+1 | 2.23E+1 | 2.98E+1 | 1.62E+1 | 1.34E+1 | 8.26E+0 | 1.50E+1 |
| **Sr** (407.771) | 0.0042 | 1.47E+2 | 1.27E+2 | 1.12E+2 | 8.17E+1 | 7.56E+1 | 9.66E+1 | 8.22E+1 | 9.06E+1 | 9.53E+1 | 7.72E+1 | 8.50E+1 | 9.53E+0 | 1.12E+2 | 1.10E+2 | 9.65E+1 | 9.48E+1 | 9.57E+1 | 1.04E+2 | 1.06E+2 | 4.61E+1 | 8.95E+1 | 6.62E+0 | 9.90E+1 |
| **Ti** (334.940) | 0.0008 | 4.83E+3 | 4.56E+3 | 4.10E+3 | 2.80E+3 | 3.01E+3 | 3.41E+3 | 3.09E+3 | 3.27E+3 | 3.18E+3 | 2.64E+3 | 2.75E+3 | 3.24E+2 | 4.04E+3 | 4.01E+3 | 3.88E+3 | 4.04E+3 | 3.96E+3 | 3.57E+3 | 2.76E+3 | 1.70E+3 | 3.42E+3 | 6.73E+1 | 3.48E+3 |
| **Tl** (190.801) | 0.0367 | 0.00E+0 | 0.00E+0 | 0.00E+0 | 0.00E+0 | 0.00E+0 | 0.00E+0 | 0.00E+0 | 0.00E+0 | 0.00E+0 | 0.00E+0 | 0.00E+0 | 0.00E+0 | 0.00E+0 | 0.00E+0 | 0.00E+0 | 0.00E+0 | 0.00E+0 | 0.00E+0 | 0.00E+0 | 0.00E+0 | 0.00E+0 | 0.00E+0 | 0.00E+0 |
| **V** (290.880) | 0.0654 | 2.04E+2 | 1.79E+2 | 1.70E+2 | 1.17E+2 | 1.24E+2 | 1.42E+2 | 1.27E+2 | 1.35E+2 | 1.34E+2 | 1.18E+2 | 1.42E+2 | 1.49E+1 | 1.50E+2 | 1.59E+2 | 1.46E+2 | 1.48E+2 | 1.48E+2 | 1.42E+2 | 1.29E+2 | 7.71E+1 | 1.36E+2 | 2.45E+0 | 1.37E+2 |
| **Zn** (206.200) | 0.0011 | 9.75E+2 | 1.55E+3 | 1.01E+3 | 7.81E+2 | 4.59E+2 | 3.71E+2 | 3.93E+2 | 4.67E+2 | 9.00E+2 | 4.83E+2 | 1.23E+3 | 1.87E+3 | 1.18E+3 | 6.77E+2 | 9.60E+2 | 1.35E+3 | 7.86E+2 | 6.56E+2 | 2.16E+3 | 8.74E+2 | 4.01E+2 | 4.09E+2 | 7.60E+2 |
| **P** (213.617) | 0.0500 | 1.52E+3 | 1.46E+3 | 1.38E+3 | 9.65E+2 | 8.07E+2 | 1.01E+3 | 7.50E+2 | 7.42E+2 | 8.46E+2 | 9.57E+2 | 2.10E+3 | 1.80E+2 | 1.28E+3 | 1.36E+3 | 1.07E+3 | 1.00E+3 | 7.78E+2 | 8.33E+2 | 2.61E+3 | 5.86E+2 | 8.76E+2 | 7.55E+1 | 7.68E+2 |
| **S** (181.975) | 0.0500 | 9.54E+3 | 8.84E+3 | 2.24E+3 | 1.24E+3 | 7.95E+2 | 8.97E+2 | 3.52E+2 | 5.93E+2 | 1.12E+3 | 3.18E+3 | 3.90E+2 | 9.07E+2 | 4.24E+3 | 1.92E+3 | 5.49E+2 | 5.30E+2 | 7.44E+2 | 7.42E+2 | 3.87E+3 | 5.31E+3 | 9.75E+2 | 1.94E+3 | 8.21E+2 |
