# Supplementary material for: Kisameet Glacial Clay: an Unexpected Source of Bacterial Diversity
Source: mBio. 2017 May 23;8(3):e00590-17. doi: 10.1128/mBio.00590-17 (PMC5442455; doi:10.1128/mBio.00590-17)
Supplement: TABLE S4 [file mbo003173310st4.docx]

**Table S4.** Physico-chemical characteristics of KC core samples.

|  | **KC35** | **1-0** | **1-4** | **1-8** | **1-12** | **1-16** | **1-20** | **1-24** | **1-28** | **2-0** | **2-36** | **3-0** | **3-4** | **3-8** | **3-12** | **3-16** | **3-20** | **3-24** | **3-28** | **4-0** | **4-16** | **5-0** | **5-28** |
| --- | --- | --- | --- | --- | --- | --- | --- | --- | --- | --- | --- | --- | --- | --- | --- | --- | --- | --- | --- | --- | --- | --- | --- |
| **pH** (aqueous suspension) | 4.30 | 6.90 | 7.21 | 8.84 | 9.47 | 9.67 | 9.76 | 9.61 | 9.50 | 7.44 | 9.31 | 8.04 | 8.35 | 9.07 | 9.33 | 9.61 | 9.43 | 9.57 | 9.48 | 8.50 | 9.66 | 7.75 | 9.50 |
| **pH** (aqueous  leachate) | 4.35 | 4.71 | 7.56 | 7.34 | 7.50 | 7.43 | 7.59 | 7.34 | 6.81 | 7.44 | 6.91 | 6.14 | 6.45 | 6.80 | 6.90 | 7.11 | 7.06 | 7.13 | 7.03 | 7.61 | 7.76 | 4.32 | 7.54 |
| **Redox** (mV) | +427 | +286 | +213 | +69.8 | +19.0 | +161.9 | +60.3 | +104.4 | +209 | +212 | +202 | +74.6 | +76.8 | +122.2 | +75.6 | +120.4 | +46.8 | +53.8 | +130.5 | +201 | +171.5 | +373 | +201 |
| **Water content**  (% wt) | 31.5 | 34.8 | 32.2 | 40.6 | 45.1 | 40.6 | 42.8 | 42.9 | 39.6 | 38.5 | 28.3 | 38.2 | 30.7 | 33.1 | 38.9 | 37.9 | 42.9 | 41.2 | 39.6 | 40.3 | 41.7 | 83.9 | 45.8 |
