# Supplementary figures and images for: Kisameet Glacial Clay: an Unexpected Source of Bacterial Diversity
Source: mBio. 2017 May 23;8(3):e00590-17. doi: 10.1128/mBio.00590-17 (PMC5442455; doi:10.1128/mBio.00590-17)

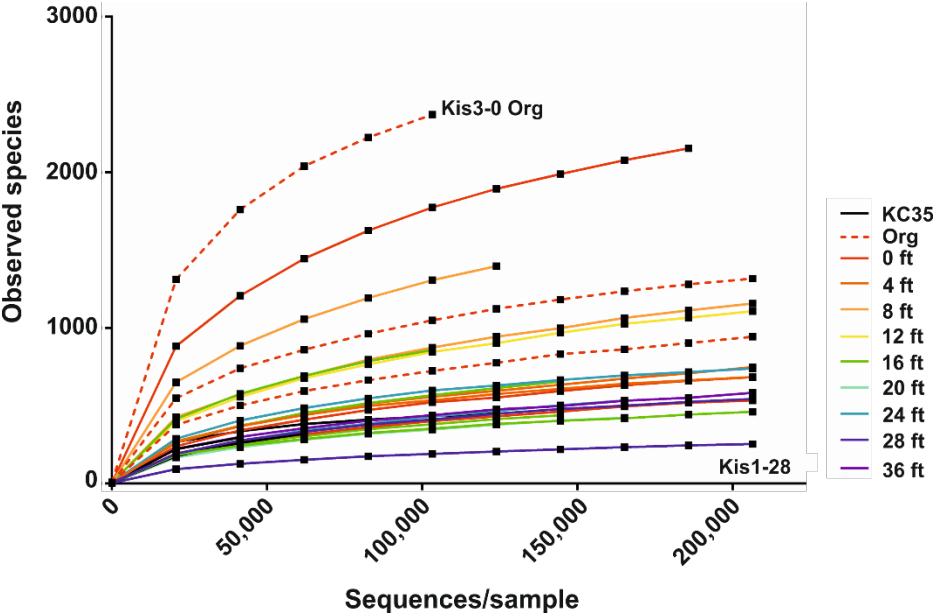

Supplement: FIG S1 [file mbo003173310sf1.pdf]

Percent of reads

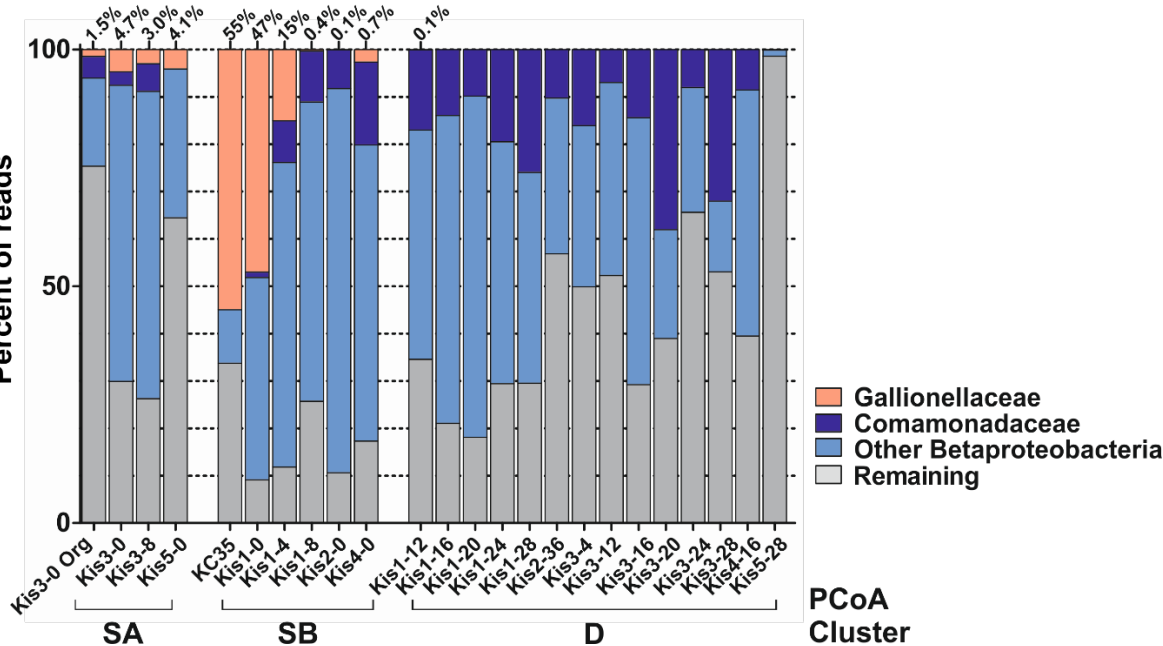

Supplement: FIG S2 [file mbo003173310sf2.pdf]

Activity vs. *E. coli* MG1655

[Fold reduction in CFU/mL at 24 h]

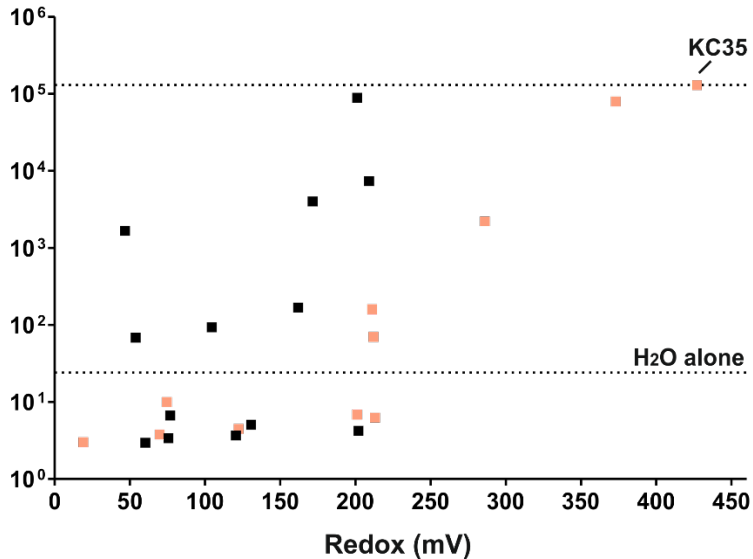

Supplement: FIG S3 [file mbo003173310sf3.pdf]

**A**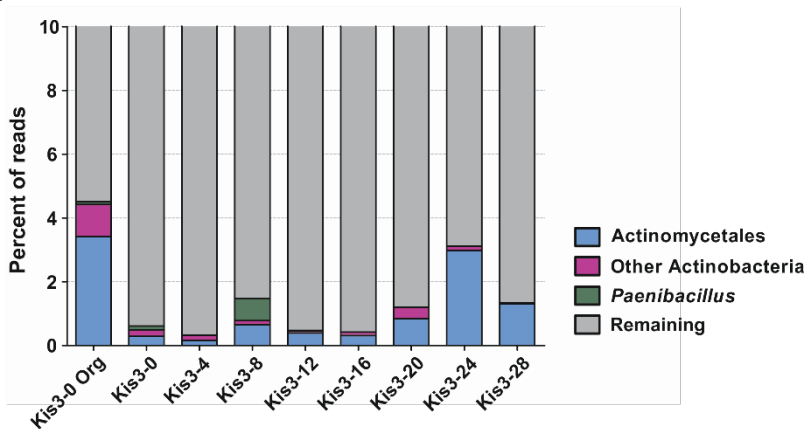**B**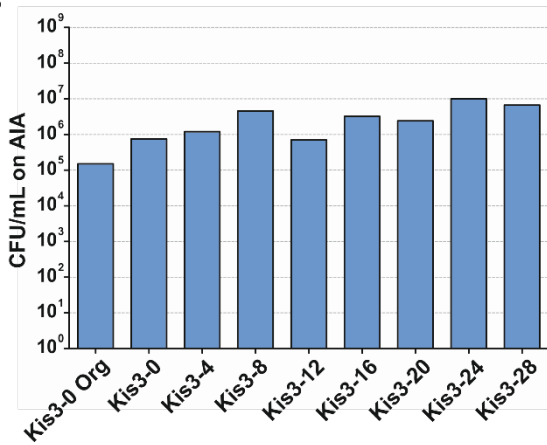

Supplement: FIG S4 [file mbo003173310sf4.pdf]
