# Supplementary material for: Kisameet Glacial Clay: an Unexpected Source of Bacterial Diversity
Source: mBio. 2017 May 23;8(3):e00590-17. doi: 10.1128/mBio.00590-17 (PMC5442455; doi:10.1128/mBio.00590-17)
Supplement: TABLE S5 [file mbo003173310st5.docx]

**Table S5.** Core bacterial community (CBC) OTUs for SA, SB, and D, as well as shared OTUs. Core OTUs were defined as those with at least one read in all samples of a group and at least 0.1% of total reads for that group.

| **CBC-SA** | | | | | | | | | | | |
| --- | --- | --- | --- | --- | --- | --- | --- | --- | --- | --- | --- |
| **OTU** | | | | **Consensus Lineage** | | | | | **% total reads in CBC-SA** | | |
| 1 | | | | Root; p__Proteobacteria; c__Betaproteobacteria; o__Burkholderiales; f__Oxalobacteraceae | | | | | 0.75 | | |
| 2 | | | | Root; p__Proteobacteria; c__Gammaproteobacteria; o__Pseudomonadales; f__Pseudomonadaceae; g__Pseudomonas | | | | | 6.76 | | |
| 3 | | | | Root; p__Proteobacteria; c__Betaproteobacteria | | | | | 26.58 | | |
| 4 | | | | Root; p__Proteobacteria; c__Betaproteobacteria; o__Gallionellales; f__Gallionellaceae; g__ | | | | | 3.61 | | |
| 6 | | | | Root; p__Proteobacteria; c__Betaproteobacteria; o__Methylophilales; f__; g__ | | | | | 4.49 | | |
| 7 | | | | Root; p__Proteobacteria; c__Betaproteobacteria; o__Methylophilales; f__Methylophilaceae; g__Methylotenera | | | | | 0.55 | | |
| 13 | | | | Root; p__Actinobacteria; c__Actinobacteria; o__Acidimicrobiales; f__; g__ | | | | | 6.80 | | |
| 17 | | | | Root; p__Proteobacteria; c__Gammaproteobacteria; o__Pseudomonadales; f__Pseudomonadaceae; g__Pseudomonas | | | | | 0.19 | | |
| 18 | | | | Root; p__Proteobacteria; c__Betaproteobacteria; o__Burkholderiales; f__Comamonadaceae | | | | | 0.30 | | |
| 27 | | | | Root; p__Proteobacteria; c__Alphaproteobacteria; o__Rhizobiales | | | | | 0.69 | | |
| 28 | | | | Root; p__Proteobacteria; c__Betaproteobacteria; o__Burkholderiales | | | | | 1.54 | | |
| 30 | | | | Root; p__Actinobacteria; c__Actinobacteria; o__Actinomycetales; f__ACK-M1; g__ | | | | | 0.56 | | |
| 34 | | | | Root; p__Proteobacteria; c__Gammaproteobacteria; o__Xanthomonadales; f__Xanthomonadaceae; g__Rhodanobacter | | | | | 0.34 | | |
| 36 | | | | Root; p__Acidobacteria; c__Acidobacteria; o__Acidobacteriales; f__Acidobacteriaceae; g__ | | | | | 2.00 | | |
| 40 | | | | Root; p__Acidobacteria; c__; o__ | | | | | 1.77 | | |
| 41 | | | | Root; p__Proteobacteria; c__Alphaproteobacteria; o__Rhodospirillales; f__Acetobacteraceae; g__Roseomonas | | | | | 0.60 | | |
| 43 | | | | Root; p__Proteobacteria; c__Gammaproteobacteria; o__Enterobacteriales; f__Enterobacteriaceae | | | | | 0.93 | | |
| 44 | | | | Root; p__Proteobacteria; c__Betaproteobacteria; o__Burkholderiales | | | | | 1.73 | | |
| 45 | | | | Root; p__Proteobacteria; c__Alphaproteobacteria; o__Rhizobiales; f__Bradyrhizobiaceae | | | | | 0.47 | | |
| 51 | | | | Root; p__Proteobacteria; c__Alphaproteobacteria; o__Rhodospirillales | | | | | 1.07 | | |
| 52 | | | | Root; p__Proteobacteria; c__Alphaproteobacteria; o__Rhodospirillales; f__Acetobacteraceae; g__Gluconobacter | | | | | 1.18 | | |
| 53 | | | | Root; p__Proteobacteria; c__Alphaproteobacteria; o__Rhizobiales; f__Beijerinckiaceae; g__ | | | | | 1.24 | | |
| 54 | | | | Root; p__Acidobacteria | | | | | 1.21 | | |
| 56 | | | | Root; p__Proteobacteria; c__Alphaproteobacteria; o__Caulobacterales; f__Caulobacteraceae; g__Caulobacter | | | | | 0.18 | | |
| 57 | | | | Root; p__Proteobacteria; c__Betaproteobacteria; o__Rhodocyclales; f__Rhodocyclaceae; g__Methyloversatilis | | | | | 0.36 | | |
| 58 | | | | Root; p__Proteobacteria; c__Betaproteobacteria | | | | | 0.89 | | |
| 59 | | | | Root; p__Proteobacteria; c__Betaproteobacteria; o__Burkholderiales; f__Oxalobacteraceae | | | | | 0.17 | | |
| 63 | | | | Root; p__Acidobacteria; c__Acidobacteria; o__Acidobacteriales; f__Acidobacteriaceae | | | | | 0.86 | | |
| 66 | | | | Root; p__Proteobacteria; c__Alphaproteobacteria; o__Rhodospirillales; f__Rhodospirillaceae; g__Telmatospirillum | | | | | 1.02 | | |
| 71 | | | | Root; p__Proteobacteria; c__Betaproteobacteria; o__Burkholderiales; f__Burkholderiaceae; g__Burkholderia | | | | | 0.83 | | |
| 74 | | | | Root; p__Acidobacteria; c__Holophagae; o__Holophagales; f__Holophagaceae | | | | | 0.62 | | |
| 77 | | | | Root; p__Proteobacteria; c__Betaproteobacteria | | | | | 0.70 | | |
| 79 | | | | Root; p__Chloroflexi; c__Dehalococcoidetes; o__NT-B4; f__; g__ | | | | | 0.39 | | |
| 81 | | | | Root; p__Proteobacteria; c__Alphaproteobacteria; o__Rhizobiales; f__Beijerinckiaceae; g__ | | | | | 0.34 | | |
| 82 | | | | Root; p__Bacteroidetes; c__Sphingobacteria; o__Sphingobacteriales; f__Sphingobacteriaceae; g__ | | | | | 0.60 | | |
| 84 | | | | Root; p__Chloroflexi; c__Dehalococcoidetes; o__NT-B4; f__; g__ | | | | | 0.47 | | |
| 89 | | | | Root; p__Firmicutes; c__Clostridia; o__Clostridiales; f__Clostridiaceae; g__Clostridium | | | | | 0.35 | | |
| 90 | | | | Root; p__Spirochaetes; c__Spirochaetes; o__Spirochaetales; f__Spirochaetaceae; g__TM3 | | | | | 0.40 | | |
| 93 | | | | Root; p__Bacteroidetes; c__Sphingobacteria; o__Sphingobacteriales; f__Sphingobacteriaceae; g__ | | | | | 0.30 | | |
| 94 | | | | Root | | | | | 0.41 | | |
| 107 | | | | Root; p__Acidobacteria; c__Solibacteres; o__Solibacterales; f__Solibacteraceae; g__CandidatusSolibacter | | | | | 0.38 | | |
| 108 | | | | Root; p__Proteobacteria; c__Deltaproteobacteria | | | | | 0.29 | | |
| 112 | | | | Root; p__Proteobacteria; c__Betaproteobacteria | | | | | 0.38 | | |
| 116 | | | | Root; p__Acidobacteria; c__Solibacteres; o__Solibacterales; f__Solibacteraceae; g__CandidatusSolibacter | | | | | 0.35 | | |
| 117 | | | | Root; p__Chloroflexi; c__Dehalococcoidetes; o__Dehalococcoidales; f__Dehalococcoidaceae; g__ | | | | | 0.18 | | |
| 119 | | | | Root; p__Acidobacteria; c__Acidobacteria; o__Acidobacteriales; f__Acidobacteriaceae; g__ | | | | | 0.22 | | |
| 120 | | | | Root; p__Acidobacteria; c__Solibacteres; o__Solibacterales; f__Solibacteraceae; g__CandidatusSolibacter | | | | | 0.29 | | |
| 124 | | | | Root; p__Proteobacteria; c__Betaproteobacteria; o__Burkholderiales; f__Oxalobacteraceae | | | | | 0.20 | | |
| 126 | | | | Root; p__Proteobacteria; c__Alphaproteobacteria; o__Rhodospirillales; f__Rhodospirillaceae | | | | | 0.27 | | |
| 146 | | | | Root; p__Acidobacteria; c__Solibacteres; o__Solibacterales; f__Solibacteraceae; g__CandidatusSolibacter | | | | | 0.20 | | |
| 150 | | | | Root; p__Proteobacteria; c__Gammaproteobacteria; o__Methylococcales; f__Crenotrichaceae; g__Crenothrix | | | | | 0.14 | | |
| 151 | | | | Root; p__Proteobacteria; c__Betaproteobacteria; o__Burkholderiales; f__Burkholderiaceae; g__Burkholderia | | | | | 0.24 | | |
| 152 | | | | Root; p__Proteobacteria; c__Betaproteobacteria; o__Neisseriales; f__Neisseriaceae; g__Silvimonas | | | | | 0.24 | | |
| 155 | | | | Root; p__Firmicutes; c__Clostridia; o__Clostridiales | | | | | 0.13 | | |
| 156 | | | | Root; p__Proteobacteria; c__Betaproteobacteria; o__Methylophilales; f__; g__ | | | | | 0.16 | | |
| 157 | | | | Root; p__Proteobacteria; c__Betaproteobacteria; o__Burkholderiales; f__Oxalobacteraceae; g__Collimonas | | | | | 0.21 | | |
| 158 | | | | Root; p__Bacteroidetes; c__Sphingobacteria; o__Sphingobacteriales; f__Sphingobacteriaceae; g__ | | | | | 0.23 | | |
| 163 | | | | Root | | | | | 0.16 | | |
| 165 | | | | Root; p__Proteobacteria; c__Betaproteobacteria | | | | | 0.17 | | |
| 166 | | | | Root; p__Acidobacteria; c__; o__; f__Koribacteraceae; g__CandidatusKoribacter | | | | | 0.16 | | |
| 167 | | | | Root; p__Chloroflexi; c__Ktedonobacteria; o__; f__; g__ | | | | | 0.18 | | |
| 170 | | | | Root; p__Proteobacteria; c__Gammaproteobacteria; o__Chromatiales; f__Sinobacteraceae; g__ | | | | | 0.16 | | |
| 173 | | | | Root; p__Proteobacteria; c__Alphaproteobacteria; o__Rhizobiales; f__Methylocystaceae; g__Methylosinus | | | | | 0.13 | | |
| 174 | | | | Root; p__Firmicutes; c__Clostridia; o__Clostridiales; f__Veillonellaceae | | | | | 0.17 | | |
| 177 | | | | Root; p__Proteobacteria; c__Alphaproteobacteria; o__Caulobacterales; f__Caulobacteraceae; g__ | | | | | 0.15 | | |
| 179 | | | | Root; p__Proteobacteria; c__Alphaproteobacteria; o__Rhizobiales; f__Hyphomicrobiaceae; g__ | | | | | 0.13 | | |
| 184 | | | | Root; p__Proteobacteria; c__Deltaproteobacteria; o__Syntrophobacterales; f__Syntrophobacteraceae; g__ | | | | | 0.15 | | |
| 187 | | | | Root | | | | | 0.16 | | |
| 190 | | | | Root; p__Proteobacteria; c__Alphaproteobacteria; o__; f__; g__ | | | | | 0.11 | | |
| 192 | | | | Root; p__Acidobacteria; c__Solibacteres; o__Solibacterales; f__Solibacteraceae; g__CandidatusSolibacter | | | | | 0.15 | | |
| 194 | | | | Root; p__Proteobacteria; c__Alphaproteobacteria; o__Rhizobiales; f__; g__ | | | | | 0.14 | | |
| 195 | | | | Root; p__Proteobacteria; c__Alphaproteobacteria; o__Rhodospirillales; f__Rhodospirillaceae | | | | | 0.10 | | |
| 196 | | | | Root; p__Acidobacteria; c__; o__ | | | | | 0.15 | | |
| 199 | | | | Root; p__Nitrospirae; c__Nitrospira; o__Nitrospirales; f__Thermodesulfovibrionaceae | | | | | 0.14 | | |
| 202 | | | | Root; p__Acidobacteria; c__Solibacteres; o__Solibacterales; f__Solibacteraceae; g__CandidatusSolibacter | | | | | 0.12 | | |
| 205 | | | | Root; p__Acidobacteria; c__; o__; f__Koribacteraceae; g__CandidatusKoribacter | | | | | 0.13 | | |
| 206 | | | | Root; p__Proteobacteria; c__Alphaproteobacteria; o__Rhizobiales; f__Hyphomicrobiaceae; g__Rhodoplanes | | | | | 0.12 | | |
| 207 | | | | Root; p__Acidobacteria; c__Solibacteres; o__Solibacterales; f__Solibacteraceae; g__CandidatusSolibacter | | | | | 0.11 | | |
| 209 | | | | Root | | | | | 0.10 | | |
| 210 | | | | Root; p__Proteobacteria; c__Alphaproteobacteria; o__Rhodospirillales; f__Acetobacteraceae; g__Acidocella | | | | | 0.12 | | |
| 218 | | | | Root | | | | | 0.12 | | |
| 231 | | | | Root; p__Acidobacteria; c__; o__ | | | | | 0.10 | | |
| 234 | | | | Root; p__Acidobacteria; c__MVS-40; o__; f__; g__ | | | | | 0.11 | | |
| 235 | | | | Root; p__Proteobacteria; c__Betaproteobacteria | | | | | 0.11 | | |
| 237 | | | | Root; p__Nitrospirae; c__Nitrospira; o__Nitrospirales; f__Thermodesulfovibrionaceae | | | | | 0.10 | | |
| **Total**:  85 OTUs | | | | **Core:** | | | | | 82.1 | | |
|  |  |  |  | **Variable:** | | | | | 17.9 | | |
| **CBC-SB** | | | | | | | | | | | |
| **OTU** | | | | **Consensus Lineage** | | | | | **% total reads in CSC-SB** | | |
| 1 | | | | Root; p__Proteobacteria; c__Betaproteobacteria; o__Burkholderiales; f__Oxalobacteraceae | | | | | 2.63 | | |
| 2 | | | | Root; p__Proteobacteria; c__Gammaproteobacteria; o__Pseudomonadales; f__Pseudomonadaceae; g__Pseudomonas | | | | | 0.55 | | |
| 3 | | | | Root; p__Proteobacteria; c__Betaproteobacteria | | | | | 9.62 | | |
| 4 | | | | Root; p__Proteobacteria; c__Betaproteobacteria; o__Gallionellales; f__Gallionellaceae; g__ | | | | | 18.49 | | |
| 5 | | | | Root; p__Proteobacteria; c__Betaproteobacteria; o__Methylophilales; f__; g__ | | | | | 9.08 | | |
| 9 | | | | Root; p__Proteobacteria; c__Betaproteobacteria | | | | | 9.63 | | |
| 16 | | | | Root; p__Proteobacteria; c__Betaproteobacteria; o__Burkholderiales; f__Comamonadaceae | | | | | 2.23 | | |
| 17 | | | | Root; p__Proteobacteria; c__Gammaproteobacteria; o__Pseudomonadales; f__Pseudomonadaceae; g__Pseudomonas | | | | | 0.61 | | |
| 18 | | | | Root; p__Proteobacteria; c__Betaproteobacteria; o__Burkholderiales; f__Comamonadaceae | | | | | 1.72 | | |
| 20 | | | | Root; p__Proteobacteria; c__Betaproteobacteria | | | | | 0.15 | | |
| 21 | | | | Root; p__Proteobacteria; c__Betaproteobacteria; o__Burkholderiales; f__Comamonadaceae | | | | | 0.47 | | |
| 27 | | | | Root; p__Proteobacteria; c__Alphaproteobacteria; o__Rhizobiales | | | | | 0.12 | | |
| 30 | | | | Root; p__Actinobacteria; c__Actinobacteria; o__Actinomycetales; f__ACK-M1; g__ | | | | | 1.33 | | |
| 33 | | | | Root; p__Proteobacteria; c__Gammaproteobacteria; o__Pseudomonadales; f__Moraxellaceae; g__Acinetobacter | | | | | 0.43 | | |
| 35 | | | | Root; p__Actinobacteria; c__Actinobacteria; o__Actinomycetales | | | | | 0.21 | | |
| 36 | | | | Root; p__Acidobacteria; c__Acidobacteria; o__Acidobacteriales; f__Acidobacteriaceae; g__ | | | | | 0.13 | | |
| 38 | | | | Root; p__Firmicutes; c__Clostridia; o__Clostridiales; f__Peptococcaceae; g__Desulfosporosinus | | | | | 0.22 | | |
| 41 | | | | Root; p__Proteobacteria; c__Alphaproteobacteria; o__Rhodospirillales; f__Acetobacteraceae; g__Roseomonas | | | | | 0.36 | | |
| 45 | | | | Root; p__Proteobacteria; c__Alphaproteobacteria; o__Rhizobiales; f__Bradyrhizobiaceae | | | | | 0.21 | | |
| 57 | | | | Root; p__Proteobacteria; c__Betaproteobacteria; o__Rhodocyclales; f__Rhodocyclaceae; g__Methyloversatilis | | | | | 0.47 | | |
| 62 | | | | Root; p__Proteobacteria; c__Betaproteobacteria | | | | | 0.54 | | |
| 70 | | | | Root; p__Firmicutes; c__Clostridia; o__Clostridiales; f__Peptococcaceae; g__Desulfosporosinus | | | | | 0.32 | | |
| 98 | | | | Root; p__Firmicutes; c__Clostridia; o__Clostridiales; f__Peptococcaceae; g__Desulfosporosinus | | | | | 0.15 | | |
| 100 | | | | Root; p__OP8; c__OP8_1 | | | | | 0.22 | | |
| 118 | | | | Root; p__Firmicutes; c__Clostridia; o__Clostridiales; f__BSV43; g__ | | | | | 0.11 | | |
| 137 | | | | Root; p__Firmicutes; c__Clostridia; o__Clostridiales; f__Veillonellaceae; g__Thermosinus | | | | | 0.13 | | |
| **Total:**  26 OTUs | | | | **Core:** | | | | | 60.1 | | |
|  |  |  |  | **Variable:** | | | | | 39.9 | | |
| **CBC-D** | | | | | | | | | | | |
| **OTU** | | | | **Consensus Lineage** | | | | | **% total reads in CBC-D** | | |
| 1 | | | | Root; p__Proteobacteria; c__Betaproteobacteria; o__Burkholderiales; f__Oxalobacteraceae | | | | | 25.48 | | |
| 2 | | | | Root; p__Proteobacteria; c__Gammaproteobacteria; o__Pseudomonadales; f__Pseudomonadaceae; g__Pseudomonas | | | | | 16.56 | | |
| 5 | | | | Root; p__Proteobacteria; c__Betaproteobacteria; o__Methylophilales; f__; g__ | | | | | 4.11 | | |
| 8 | | | | Root; p__Proteobacteria; c__Betaproteobacteria; o__Burkholderiales; f__Comamonadaceae; g__Comamonas | | | | | 7.04 | | |
| 10 | | | | Root; p__Proteobacteria; c__Gammaproteobacteria; o__Pseudomonadales; f__Moraxellaceae; g__Acinetobacter | | | | | 3.85 | | |
| 11 | | | | Root; p__Proteobacteria; c__Gammaproteobacteria; o__Xanthomonadales; f__Xanthomonadaceae | | | | | 3.81 | | |
| 17 | | | | Root; p__Proteobacteria; c__Gammaproteobacteria; o__Pseudomonadales; f__Pseudomonadaceae; g__Pseudomonas | | | | | 1.48 | | |
| 32 | | | | Root; p__Proteobacteria; c__Gammaproteobacteria; o__Pseudomonadales; f__Pseudomonadaceae; g__Pseudomonas | | | | | 0.80 | | |
| 35 | | | | Root; p__Actinobacteria; c__Actinobacteria; o__Actinomycetales | | | | | 0.58 | | |
| 43 | | | | Root; p__Proteobacteria; c__Gammaproteobacteria; o__Enterobacteriales; f__Enterobacteriaceae | | | | | 0.25 | | |
| 46 | | | | Root; p__Proteobacteria; c__Gammaproteobacteria; o__Enterobacteriales; f__Enterobacteriaceae; g__Serratia | | | | | 0.46 | | |
| 55 | | | | Root; p__Proteobacteria; c__Gammaproteobacteria; o__Pseudomonadales; f__Pseudomonadaceae; g__Pseudomonas | | | | | 0.28 | | |
| 56 | | | | Root; p__Proteobacteria; c__Alphaproteobacteria; o__Caulobacterales; f__Caulobacteraceae; g__Caulobacter | | | | | 0.26 | | |
| 60 | | | | Root; p__Chloroflexi; c__Anaerolineae | | | | | 0.33 | | |
| 68 | | | | Root; p__OP9; c__JS1; o__SB-45; f__; g__ | | | | | 0.25 | | |
| 91 | | | | Root; p__Proteobacteria; c__Deltaproteobacteria; o__; f__; g__ | | | | | 0.12 | | |
| 92 | | | | Root | | | | | 0.14 | | |
| 103 | | | | Root; p__Proteobacteria; c__Gammaproteobacteria; o__Pseudomonadales; f__Moraxellaceae; g__Acinetobacter | | | | | 0.12 | | |
| **Total:**  18 OTUs | | | | **Core:** | | | | | 65.9 | | |
|  |  |  |  | **Variable:** | | | | | 34.1 | | |
| **Shared** | | | | | | | | | | | |
| **OTU** | **Consensus Lineage** | | | **All** | **SA + SB** | **SB + D** | | | **SA + D** | | |
| 1 | p__Proteobacteria; c__Betaproteobacteria; o__Burkholderiales; f__Oxalobacteraceae | | | * | * | * | | | * | | |
| 2 | p__Proteobacteria; c__Gammaproteobacteria; o__Pseudomonadales; f__Pseudomonadaceae; g__Pseudomonas | | | * | * | * | | | * | | |
| 3 | p__Proteobacteria; c__Betaproteobacteria | | |  | * |  | | |  | | |
| 4 | p__Proteobacteria; c__Betaproteobacteria; o__Gallionellales; f__Gallionellaceae; g__ | | |  | * |  | | |  | | |
| 5 | p__Proteobacteria; c__Betaproteobacteria; o__Methylophilales; f__; g__ | | |  |  | * | | |  | | |
| 17 | p__Proteobacteria; c__Gammaproteobacteria; o__Pseudomonadales; f__Pseudomonadaceae; g__Pseudomonas | | | * | * | * | | | * | | |
| 18 | p__Proteobacteria; c__Betaproteobacteria; o__Burkholderiales; f__Comamonadaceae | | |  | * |  | | |  | | |
| 27 | p__Proteobacteria; c__Alphaproteobacteria; o__Rhizobiales | | |  | * |  | | |  | | |
| 35 | p__Actinobacteria; c__Actinobacteria; o__Actinomycetales | | |  |  | * | | |  | | |
| 41 | p__Proteobacteria; c__Alphaproteobacteria; o__Rhodospirillales; f__Acetobacteraceae; g__Roseomonas | | |  | * |  | | |  | | |
| 43 | p__Proteobacteria; c__Gammaproteobacteria; o__Enterobacteriales; f__Enterobacteriaceae | | |  |  |  | | | * | | |
| 45 | p__Proteobacteria; c__Alphaproteobacteria; o__Rhizobiales; f__Bradyrhizobiaceae | | |  | * |  | | |  | | |
| 56 | p__Proteobacteria; c__Alphaproteobacteria; o__Caulobacterales; f__Caulobacteraceae; g__Caulobacter | | |  |  |  | | | * | | |
| 57 | p__Proteobacteria; c__Betaproteobacteria; o__Rhodocyclales; f__Rhodocyclaceae; g__Methyloversatilis | | |  | * |  | | |  | | |
|  | **Shared OTUs:** | | | 3 | 10 | 5 | | | 5 | | |
