# Supplementary material for: Kisameet Glacial Clay: an Unexpected Source of Bacterial Diversity
Source: mBio. 2017 May 23;8(3):e00590-17. doi: 10.1128/mBio.00590-17 (PMC5442455; doi:10.1128/mBio.00590-17)
Supplement: TEXT S1 [file mbo003173310s1.docx]

**Supplementary methods**

**Next-generation sequencing of KC 16S amplicons and sequence data analysis**. Briefly, 341F and 518R 16S rRNA primers (1), modified for adaptation to the Illumina (San Diego, CA) Miseq platform and including the addition of 6 bp unique barcodes to the reverse primer allowing for multiplex amplification, were used to amplify the 16S rDNA region by PCR. PCR amplification, separation by gel electrophoresis, and gel extraction steps were completed as described in (2), with some modifications as described in (3, 4). Briefly, the amount of primer used was decreased to 5 pmol each, a *Taq* polymerase (Life Technologies, Carlsbad, CA) was used for amplification, and the cycling times were changed to 30 seconds for each step. Products were then sequenced using the Illumina MiSeq platform (paired-end, 250 bp) at the McMaster Genomics Facility.

For sequence data analysis, Cutadapt (5) was used to trim any reads surpassing the length of the V3 region. Resulting paired-end sequences were aligned with PANDAseq (6). Following trimming, quality control, and removal of doubleton/singleton reads, we had approximately 4.91 million reads, with a range of 46,471 to 333,878 (mean 196,314) (**Table S1**). Operational taxonomic units (OTUs) were picked using AbundantOTU+ (7) with a clustering threshold of 97%. Taxonomy was assigned using the Ribosomal Database Project classifier (8), with a minimum confidence cutoff of 0.8 (QIIME default) against the Greengenes (February 4, 2011 release) reference database to the genus level (9). Samples were rarified to 46,471 sequences for all subsequent analyses. Summaries of the relative abundances of taxonomies were computed using Quantitative Insights Into Microbial Ecology (QIIME) (10). To determine alpha-diversity (i.e. within core sample communities), the number of observed OTUs (community richness) and Shannon index (community diversity) (11) was calculated using QIIME. Beta-diversity (differences between core sample communities) was calculated with the unweighted UniFrac distance with samples rarefied to 46,471 sequences. Clustering based on distance was shown with principal coordinate analysis (PCoA).

**Preparation of aqueous leachates.** Aqueous leachates were prepared from wet clay in double-distilled, deionized, autoclaved water with a pH of approximately 6.9 at a concentration of 500 mg/mL (50% w/v). The water content of each clay sample was determined by measuring the mass lost after heat-drying (**Table S4**), and was found to be between 30 – 45 % for all samples except Kis5-0, which was composed entirely of organic material and had a high percentage of water (~85%). Ten grams of wet clay was weighed and suspended in water of an appropriate volume for a final concentration of 50% (~20 mL) in a flask, and incubated sealed at room temperature, for 24 h with shaking at 200 rpm. Suspensions were then centrifuged at 20,000 rpm for 2 h to remove particulate matter. Leachates were further clarified by filtration of the supernatant through a 0.22 μM-cutoff filter (Millipore). The resulting leachates were stored in the dark at 4 ^°^C until testing.

**Mineralogical composition by XRD.** The samples were reduced to the optimum grain-size range for quantitative X-ray analysis (<10 μm) by grinding under ethanol in a vibratory McCrone Micronising Mill for 7 minutes. Continuous-scan X-ray powder-diffraction data were collected over a range 3-80°2θ with CoKa radiation on a Bruker D8 Focus Bragg-Brentano diffractometer equipped with an Fe monochromator foil, 0.6 mm (0.3°) divergence slit, incident- and diffracted-beam Soller slits and a LynxEye detector. The long fine-focus Co X-ray tube was operated at 35 kV and 40 mA, using a take-off angle of 6°. The X-ray diffractogram was analyzed using the International Centre for Diffraction Database PDF-4 and Search-Match software by Bruker. X-ray powder-diffraction data of the samples were refined with Rietveld program Topas 4.2 (Bruker AXS). The results of quantitative phase analysis by Rietveld refinements are summarized in **Table S2**. These amounts represent the relative amounts of crystalline phases normalized to 100% amorphous-free.

**Elemental composition by ICP-OES.** Elemental concentrations were determined by inductively coupled plasma optical emission spectrometry (ICP-OES) after acid digestion, according to method 3050B (12). Values for aqueous suspensions and bulk clay are displayed in **Table S3 & S4**, respectively.

**Measurement of pH and redox.** Measurements of pH, redox potential, and electrical conductivity were performed based on standard methods and are summarized in **Table S3**. Measurement of KC pH was performed using either an equilibrated suspension of 1 g clay in 10 mL distilled H_2_O (measured immediately) or aqueous leachates using a VWR-SB20 pH meter. The redox potential was measured using a Beckman Phi44 pH meter with means for temperature compensation (with redox probe) (USEPA method 9045D) (12).

**Antibacterial activity of core samples.** Quantification of antibacterial activity was performed essentially as previously described (13). The wildtype *Escherichia coli* K12 strain MG1655 (Bachman 1972; Davies lab strain collection), grown aerobically at 37 °C in Luria-Bertani broth (LB) was used as a test strain. Ten milligrams of dried autoclaved clay was suspended in one milliliter of sterile H_2_O. Log-phase *E. coli* (OD_600_ ~0.4) were washed twice with sterile H_2_O, and used to inoculate each tube with approximately 10^7^ CFU/mL of bacteria. Clay-bacteria suspensions were incubated with shaking at 37 ^°^C for 24 h, serially diluted and plated on LB agar for CFU enumeration. Results show the mean fold-reduction in CFUs compared to the inoculum for four independent trials. A suspension of KC35, a highly active sample of KC (13), was used as a positive control, and sterile water was used as a negative control. To control for the low pH of some KC suspensions, viability of *E. coli* in 100 mM phosphate buffer, pH 4.3 (the pH of KC35 aqueous leachates) was determined.

1. Muyzer G, de Waal EC, Uitterlinden AG. 1993. Profiling of complex microbial populations by denaturing gradient gel electrophoresis analysis of polymerase chain reaction-amplified genes coding for 16S rRNA. Appl Environ Microbiol 59:695-700.

2. Bartram AK, Lynch MD, Stearns JC, Moreno-Hagelsieb G, Neufeld JD. 2011. Generation of multimillion-sequence 16S rRNA gene libraries from complex microbial communities by assembling paired-end illumina reads. Appl Environ Microbiol 77:3846-52.

3. Whelan FJ, Verschoor CP, Stearns JC, Rossi L, Luinstra K, Loeb M, Smieja M, Johnstone J, Surette MG, Bowdish DM. 2014. The loss of topography in the microbial communities of the upper respiratory tract in the elderly. Ann Am Thorac Soc 11:513-21.

4. Stearns JC, Davidson CJ, McKeon S, Whelan FJ, Fontes ME, Schryvers AB, Bowdish DM, Kellner JD, Surette MG. 2015. Culture and molecular-based profiles show shifts in bacterial communities of the upper respiratory tract that occur with age. ISME J 9:1268.

5. Martin M. 2011. Cutadapt removes adapter sequences from high-throughput sequencing reads. EMBnet J 17:10–12.

6. Masella AP, Bartram AK, Truszkowski JM, Brown DG, Neufeld JD. 2012. PANDAseq: paired-end assembler for illumina sequences. BMC Bioinformatics 13:31.

7. Ye Y. 2011. Identification and Quantification of Abundant Species from Pyrosequences of 16S rRNA by Consensus Alignment. Proceedings (IEEE Int Conf Bioinformatics Biomed) 2010:153-157.

8. Wang Q, Garrity GM, Tiedje JM, Cole JR. 2007. Naive Bayesian classifier for rapid assignment of rRNA sequences into the new bacterial taxonomy. Applied and Environmental Microbiology 73:5261-5267.

9. DeSantis TZ, Hugenholtz P, Larsen N, Rojas M, Brodie EL, Keller K, Huber T, Dalevi D, Hu P, Andersen GL. 2006. Greengenes, a chimera-checked 16S rRNA gene database and workbench compatible with ARB. Applied and Environmental Microbiology 72:5069-5072.

10. Caporaso JG, Kuczynski J, Stombaugh J, Bittinger K, Bushman FD, Costello EK, Fierer N, Pena AG, Goodrich JK, Gordon JI, Huttley GA, Kelley ST, Knights D, Koenig JE, Ley RE, Lozupone CA, McDonald D, Muegge BD, Pirrung M, Reeder J, Sevinsky JR, Tumbaugh PJ, Walters WA, Widmann J, Yatsunenko T, Zaneveld J, Knight R. 2010. QIIME allows analysis of high-throughput community sequencing data. Nature Methods 7:335-336.

11. Shannon CE, Weaver W. 1949. The mathematical theory of communication. University of Illinois Press, Urbana,.

12. USEPA. 2007. Test methods for evaluating solid wastes, SW 846 USEPA Office of Solid Waste, 3rd Edition (1986) including Update I (1992) Update II, IIA, IIB (1993-1995) Update III, IIIA, IIIB (1996-2004) and Update IV (2007) (EPA 3000, 9000 series Methods.

13. Behroozian S, Svensson SL, Davies J. 2016. Kisameet Clay Exhibits Potent Antibacterial Activity against the ESKAPE Pathogens. MBio 7:e01842-15.
